# Comparative Preclinical Evaluation of the Tumor-Targeting Properties of Radioiodine and Technetium-Labeled Designed Ankyrin Repeat Proteins for Imaging of Epidermal Growth Factor Receptor Expression in Malignant Tumors

**DOI:** 10.3390/ijms262110609

**Published:** 2025-10-31

**Authors:** Mariia Larkina, Gleb Yanovich, Lutfi Aditya Hasnowo, Ruslan Varvashenya, Feruza Yuldasheva, Maria Tretyakova, Evgenii Plotnikov, Roman Zelchan, Alexey Schulga, Elena Konovalova, Rustam Ziganshin, Mikhail V. Belousov, Vladimir Tolmachev, Sergey M. Deyev

**Affiliations:** 1 Research Centrum for Oncotheranostics, Research School of Chemistry and Applied Biomedical Sciences, Tomsk Polytechnic University, 634050 Tomsk, Russia; marialarkina@mail.ru (M.L.); sonne_gleb@mail.ru (G.Y.); mr.varvashenya@mail.ru (R.V.); feruza.yuldasheva.2016@gmail.com (F.Y.); trremar@mail.ru (M.T.); plotnikov.e@mail.ru (E.P.); r.zelchan@yandex.ru (R.Z.); schulga@gmail.com (A.S.); elena.ko.mail@gmail.com (E.K.); mvb63@mail.ru (M.V.B.); biomem@mail.ru (S.M.D.); 2 Department of Pharmaceutical Analysis, Siberian State Medical University, 634050 Tomsk, Russia; 3 Polytechnic Institute of Nuclear Technology, National Research and Innovation Agency of Indonesia, Yogyakarta 55281, Indonesia; lutf006@brin.go.id; 4 Department of Nuclear Medicine, Cancer Research Institute, Tomsk National Research Medical Center, Russian Academy of Sciences, 634009 Tomsk, Russia; 5 Molecular Immunology Laboratory, Shemyakin & Ovchinnikov Institute of Bioorganic Chemistry, Russian Academy of Sciences, 117997 Moscow, Russia; 6 Collective Use Center “Bioorganic”, Shemyakin & Ovchinnikov Institute of Bioorganic Chemistry, Russian Academy of Sciences, 117997 Moscow, Russia; ziganshin@mail.ru; 7 Department of Immunology, Genetics and Pathology, Uppsala University, 75185 Uppsala, Sweden

**Keywords:** EGFR imaging, DARPin E01, radiolabeling, technetium-99m, iodine-123, residualizing label, non-residualizing label, tumor targeting, biodistribution, SPECT imaging

## Abstract

Radionuclide molecular imaging of epidermal growth factor receptor (EGFR) expression might permit the selection of patients for EGFR-targeting therapies. Designed ankyrin repeat protein (DARPin) E01 with a high affinity to the ectodomain III of the EGFR is a possible EGFR imaging probe. The goal of this study was to evaluate the potential of radiolabeled DARPin E01 for in vivo imaging of EGFR. DARPin E01 containing the (HE)_3_-tag was site-specifically labeled with a residualizing ^99m^Tc (using ^99m^Tc]Tc(CO)_3_). Two methods providing non-residualizing ^123^I labels, direct electrophilic radioiodination and indirect radioiodination using [^123^I]I-*para*-iodobenzoate (PIB), were tested. [^99m^Tc]Tc-(HE)_3_-E01 and [^123^I]I-(HE)_3_-E01-PIB preserved specific binding to EGFR-expressing cells and affinity in the single-digit nanomolar range. Direct labeling with ^123^I resulted in a substantial loss of binding. In vitro cellular processing studies showed that both [^99m^Tc]Tc-(HE)_3_-E01 and [^123^I]I-(HE)_3_-E01-PIB had rapid binding and relatively slow internalization. Evaluation of [^99m^Tc]Tc-(HE)_3_-E01 biodistribution in normal CD1 mice showed that its hepatic uptake was non-saturable, suggesting that this tracer does not bind to murine EGFR. A side-by-side comparison of biodistribution and tumor targeting of [^99m^Tc]Tc-(HE)_3_-E01 and [^123^I]I-(HE)_3_-E01-PIB was performed in Nu/j mice bearing EGFR-positive A-431 and EGFR-negative Ramos human cancer xenografts. Both radiolabeled DARPins demonstrated EGFR-specific tumor uptake. However, [^123^I]I-(HE)_3_-E01-PIB had appreciably lower uptake in normal organs compared to [^99m^Tc]Tc-(HE)_3_-E01, which provided significantly (*p* < 0.05) higher tumor-to-organ ratios. Gamma-camera imaging confirmed that [^123^I]I-(HE)_3_-E01-PIB demonstrated a higher imaging contrast in preclinical models than [^99m^Tc]Tc-(HE)_3_-E01. In conclusion, DARPin (HE)_3_-E01 labeled using a non-residualizing [^123^I]I-*para*-iodobenzoate (PIB) label is the preferred radiotracer for in vivo imaging of EGFR expression in cancer.

## 1. Introduction

In vivo visualization of cell-surface receptor overexpression in malignant tumors has the potential to identify specific molecular targets that can be utilized to treat tumors in a personalized manner [1]. There has been a growing interest in the use of in vivo imaging techniques for the assessment of receptor tyrosine kinases (RTK) expression [2]. The epidermal growth factor receptor (EGFR, ErbB1, HER1) is a transmembrane protein of the tyrosine kinase receptor family. EGFR activation generates signaling that may contribute to cell proliferation, increased motility, and suppression of apoptosis [3]. Several anti-EGFR monoclonal antibodies (cetuximab, panitumumab, nimotuzumab, necitumumab) have been approved by the FDA for the therapy of EGFR-expressing tumors [4]. The receptor’s expression level is among the potential determinants for the response to such therapy [5].

The correlation between EGFR expression and cancer prognosis was studied in a retrospective review of EGFR studies. According to reports, EGFR expression level is a significant prognostic indicator for patients with head and neck carcinomas [6,7], ovarian [8], cervical, bladder [9,10], and esophageal cancers [11]. The prognostic value is somewhat lower for gastric, breast [12], endometrial, and colorectal tumors [13], and for non-small cell lung cancer [11,14,15]. Hence, the identification of EGFR expression levels in tumors may provide significant diagnostic insights that can impact patient management [4].

Targeted radionuclide imaging of EGFR expression offers several potential advantages compared to biopsy analysis. This is primarily due to the reduced possibility of false-negative results caused by several factors such as sampling errors, expression heterogeneity, discordance of EGFR expression between primary tumors and metastases, inadequate procedures and antibodies, and alterations in EGFR status during therapy [16]. Substantial efforts were devoted to the development of EGFR imaging radiotracers [16]. The majority of previously developed radiotracers for radionuclide molecular imaging of tumors were based on monoclonal antibodies (mAb) [17,18,19]. Nevertheless, there are evident concerns primarily related to the size of the antibodies. These include the sluggish elimination of mAbs from the bloodstream, resulting in a diminished signal-to-background ratio during imaging and decreased sensitivity [18]. Additionally, the extravasation and diffusion rates of antibodies in the tumor interstitium are slow [20]. This necessitates several days between antibody administration and imaging. Furthermore, some accumulation of full-length IgG antibodies in tumors is non-specific, attributable to the enhanced permeability and retention (EPR) effect commonly observed for large macromolecules [21,22]. The tumor uptake of unspecific antibodies might be as high as 50% of specific ones. This is associated with a risk of false-positive diagnoses [23,24].

The use of smaller radiolabeled fragments of cetuximab as imaging agents has significantly enhanced the contrast of EGFR imaging and enabled a reduction of the time interval between the administration of the radiotracer and the imaging session [25,26,27]. In recent years, there has been a growing recognition of the potential of small engineered scaffold proteins (ESPs) as viable alternatives to antibodies in cancer treatment and detection. These proteins offer several distinct benefits, including high thermal and chemical stability, the ability to be produced in prokaryotic systems, a high degree of modularity, and enhanced capabilities for extravasation and tissue penetration [28]. When comparing engineered scaffold proteins to monoclonal antibodies, it is evident that ESPs have more advantageous characteristics for imaging purposes. This is primarily attributed to their smaller size, which facilitates swift tumor localization and quick reduction of background activity associated with blood, owing to efficient renal clearance [22,29]. The anti-EGFR ESP, affibody molecules, demonstrated successful imaging of EGFR in preclinical tumor models [22]. However, our experience with scaffold proteins targeting HER2 [22] shows that the different scaffolds may have dissimilar uptake in normal tissues, most likely due to different off-target interactions. This causes appreciable variations in tumor-to-organ radios and, therefore, in the contrast in the case of radionuclide imaging. Due to the complexity of in vivo off-target interactions, the distribution is difficult to predict. Therefore, it would be important to explore other ESPs for the imaging of EGFR to select the best one.

Designed Ankyrin Repeat Proteins (DARPins) are a class of non-immunoglobulin scaffolds constructed using a series of closely arranged repeats, typically consisting of 33 amino acid residues. A single protein can include up to 29 consecutive repeats, with each repeat including a structural unit that consists of a β-turn followed by two antiparallel α-helices [30]. Typically, ankyrin repeat domains consist of four to six repeats, resulting in a solenoid structure with a right-handed conformation having a continuous hydrophobic core and a large solvent-accessible surface [30,31]. Randomization of surface amino acids enables the development of libraries featuring a groove-like target-binding surface with efficient folding properties and high solubility [32,33]. Similar to other ESPs, DARPins possess several beneficial characteristics compared to antibodies and antibody fragments for in vivo targeting [34,35,36,37]. Importantly, recombinant production in prokaryotic cells offers several advantages over antibodies, including increased yields and reduced manufacturing expenses [30]. Clinical studies demonstrated that radiolabeled DARPins can be used for highly specific visualization of expression of other molecular targets, HER2 [38] and EpCAM [39] in malignant tumors. These imaging probes demonstrated rapid clearance from blood (elimination half-lives 3.5–3.8 h and 2.3 h for HER2- and EpCAM-targeting DARPins, respectively), which resulted in low background and provided high-contrast imaging 2–4 h after injection. No adverse effects were observed in these studies. Some such effects were detected in a clinical therapy study when multiple injections of high doses (up to 12 mg/kg) of a DARPin MP250 with an extended residence in blood were performed repeatedly [40]. In that study, a formation of antidrug antibodies was detected; however, there was no effect on the pharmacokinetics of DARPins.

Phage display methodology enabled the selection of several DARPins binding to EGFR with subnanomolar affinity [41]. DARPin E01 had the highest affinity to EGFR (apparent equilibrium dissociation constant (K_D_) of 0.5 nM) and was not prone to spontaneous oligomerization. E01 binds to the ectodomain III of the EGFR, and its epitope partially overlaps with the epitope of the natural ligand, epidermal growth factor (EGF), and the therapeutic anti-EGFR antibody cetuximab [41,42]. Moreover, it has been demonstrated that DARPin E01 can suppress EGFR phosphorylation [33,43]. Such levels of specificity and affinity render E01 a potential targeting vector for radionuclide visualization of EGFR.

A notable obstacle encountered in imaging EGFR in tumors is the significant expression of EGFR on hepatocytes, which creates an inherent barrier for blood-borne imaging agents [44]. However, it was shown that EGFR on hepatocytes can be partially saturated by adjusting the quantity of injected targeting protein, allowing radiolabeled radiotracers to cross the “liver barrier” [16,45]. Still, the hepatic absorption of radiolabeled tracers through interaction with EGFR on hepatocytes is not the sole mechanism involved in the hepatic uptake. Studies with affibody molecules demonstrated that the hepatic uptake is significantly influenced by the physicochemical features of the combination of a chelator or prosthetic group with a radionuclide [22,45]. Determining the optimal radiolabeling method for ESPs is inherently more complex than for antibodies due to the smaller size of ESPs. The physicochemical characteristics of the ESPs undergo significant changes as a result of radiolabeling. The selection of a labeling method has the potential to influence several aspects of an engineered targeting moiety’s interaction with its intended molecular target. These include the strength of binding, how the targeting moiety is processed inside cells, the extent to which radiometabolites are retained within malignant cells following internalization, and the occurrence of unintended interactions with non-malignant tissues [46]. Additionally, the primary excretion pathway of the imaging radiotracer, its absorption and retention in excretory organs, and the excretion of its radiometabolites may be altered [20].

Our earlier studies have demonstrated that the substitution of the hexahistidine (H_6_) tag with a histidine-glutamate-histidine-glutamate-histidine-glutamate (HEHEHE- or (HE)_3_) tag improves the biodistribution of DARPins by reducing their uptake in the liver. Vorobyeva et al. have shown that using the (HE_3_)-tag instead of the H_6_ tag for labeling HER2-imaging DARPin G3 with [^99m^Tc]Tc(CO)_3_ resulted in a threefold reduction in hepatic uptake [47]. The use of this tag for labeling of the EpCAM-targeting DARPin Ec1 also reduced the hepatic uptake of Ec1 2.3-fold [46]. Both G3 and Ec1 labeled using (HE)_3_ tags showed appropriate in vivo distribution in clinical studies [38,39]. Therefore, the (HE)_3_ tag was selected for labeling of E01 with ^99m^Tc. This label is residualizing, i.e., it is retained inside the cell after internalization. This enables identification of organs, where the radiolabeled protein was internalized and catabolized. In the case of a rapid internalization of radiolabeled targeting proteins by malignant cells, the use of a residualizing label increases tumoral retention of activity. Radiometabolites of a non-residualizing (e.g., radioiodine) label diffuse from cells after internalization and proteolysis in lysosomes. The use of non-residualizing radioiodine labels significantly reduces uptake in normal tissues, particularly in the liver and kidneys, where DARPin appears to be rapidly internalized [46,48]. If the targeting protein is rapidly internalized by cancer cells, the tumor retention of activity might be poor. In the case of HER2- and EpCAM- targeting DARPins, the internalization by cancer cells was slow, and the tumor retention was good. It is important to acknowledge that the straight translation of findings from one DARPin-based radiotracer to another may be overly optimistic due to potentially significant differences between various DARPins. DARPins may contain a different number of repeats [46]. This prompted testing the same labelling approach to evaluate whether it would impart positive characteristics to other DARPins.

The goal of this study was to evaluate the potential of DARPin E01 containing (HE)_3_ tag ((HE)_3_-E01) for visualization of EGFR in vivo using single-photon emission computed tomography (SPECT). For this purpose, (HE)_3_-E01 was site-specifically labeled with technetium-99m using technetium tricarbonyl (variant [^99m^Tc]Tc-(HE)_3_-E01). Alternatively, (HE)_3_-E01 was labeled with iodine-123. Two radioiodination methods were evaluated: direct oxidative iodination, placing the label on tyrosines of proteins (variant [^123^I]I-(HE)_3_-E01), and indirect labeling by conjugation of [^123^I]I-para-iodobenzoate to lysines (valiant [^123^I]I-(HE)_3_-E01-PIB). The binding specificity of all variants was evaluated in vitro using the EGFR-expressing cell lines. The promising variants [^99m^Tc]Tc-(HE)_3_-E01 and [^123^I]I-(HE)_3_-E01-PIB were further compared using affinity, cellular processing, biodistribution assays, and imaging experiments.

## 2. Results

### 2.1. Protein Production and Characterization

DARPin E01 variant with the amino acid sequence histidine-glutamate-histidine-glutamate-histidine-glutamate (HE)_3_ at the N-terminus was produced using *E. coli*. The molecular weight of the DARPin (HE)_3_-E01 was confirmed by mass spectrometry (Figures S1–S3 in Supplementary Information). The obtained value demonstrated excellent agreement between the theoretical and found molecular weights (18,260.25 Da and 18,260.29 Da, respectively).

### 2.2. Radiolabeling

DARPin E01 was successfully site-specifically labeled with [^99m^Tc][Tc(CO)_3_(H_2_O)_3_]^+^ using a triple histidine-glutamate tag, (HE)_3_-tag, as a chelator to provide a residualizing label. The construct was designated as [^99m^Tc]Tc-(HE)_3_-E01. The radiochemical yield of [^99m^Tc]Tc-(HE)_3_-E01 was not significantly different between the protocols with and without an intermediate histidine challenge, 90 ± 2% (*n* = 3).

The synthesis of N-succinimidyl 4-(trimethylstannyl)benzoate was carried out as outlined in Figure S4 (in Supplementary Information). The prosthetic group was produced from the corresponding iodobenzoic acid in two steps via palladium-catalyzed stannylation with subsequent introduction of the succinimide group by using 1-ethyl-3-(3-dimethylaminopropyl)carbodiimide hydrochloride. This study differed from the approach of [49], which used di(N-succinimidyl) carbonate as the precursor of succinimide, whereas the current research uses N-hydroxysuccinimide. Based on this method, the yield of the prosthetic group was 88%.

**Figure 1:**
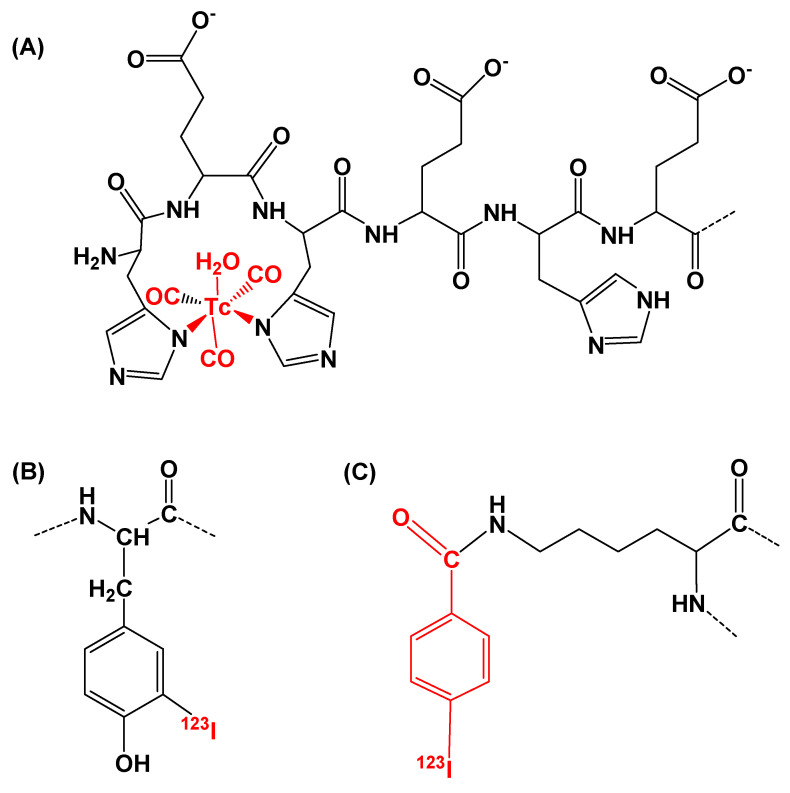
The overview of the structures of (**A**) [^99m^Tc]Tc(CO)_3_^+^ with the (HE)_3_ tag as a chelator at N-terminus of the DARPin E01, (**B**) ^123^I-directly labeled phenolic ring of one of four DARPin E01 tyrosines, and (**C**) [^123^I]I-PIB conjugated to the N-terminal or one of eight lysine amino groups of DARPin E01. Atoms and groups of atoms incorporated in the protein structure by labeling are marked in red.

For indirect site-unspecific radioiodination, the N-succinimidyl-*p*-(trimethylstannyl)benzoate prosthetic group was iodinated first, and then *para*-[^123^I]iodobenzoate (designated [^123^I]I-PIB) was conjugated to DARPin (HE)_3_-E01. Radiochemical yield of [^123^I]I-(HE)_3_-E01-PIB was 15 ± 6% (*n* = 4). For direct site-unspecific radioiodination, ^123^I was incorporated into the phenolic ring of tyrosines in the DARPin (HE)_3_-E01. The radiochemical yield for [^123^I]I-(HE)_3_-E01 was 83 ± 1% (*n* = 2). After purification using a NAP-5 size-exclusion column, the radiochemical purity for all variants was close to 100%. Characteristics of the labeling procedures and labeled DARPins are presented in Table 1. The overview of the radiolabel’s structures is presented in Figure 1.

### 2.3. In Vitro Studies

#### 2.3.1. Specificity Test

**Figure 2:**
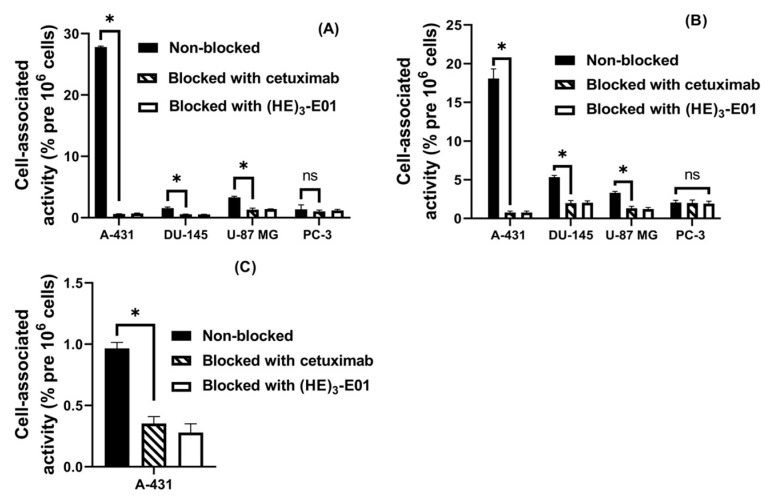
In vitro binding specificity of (**A**) [^99m^Tc]Tc-(HE)_3_-E01 and (**B**) [^123^I]I-(HE)_3_-E01-PIB, and (**C**) [^123^I]I-(HE)_3_-E01 to EGFR-expressing cells. To block receptors in control groups, a *100*-fold excess of cetuximab and unlabeled (HE)_3_-E01 was added. Data are presented as the mean of three samples ± standard deviation (SD). An asterisk indicates that the differences between groups were significant (*p* ˂ 0.05), “ns” indicates that the differences between groups were not significant (*p* > 0.05).

Four EGFR-expressing cell lines with varying levels of EGFR expression, A-431, DU-145, U-87 MG, and PC3, were used for cell studies. Pre-saturation of the EGFR by both an excess of cetuximab and of unlabeled (HE)_3_-E01 reduced significantly (*p* < 0.05, unpaired *t*-test) the level of cell-associated activity of [^99m^Tc]Tc-(HE)_3_-E01 and [^123^I]I-(HE)_3_-E01-PIB to EGFR-expressing A-431, DU-145, and U-87 MG cell lines (Figure 2). There was no significant difference (*p* > 0.05 in unpaired *t*-test) in binding to PC-3 with low EGFR expression with or without the receptor pre-saturation. This test demonstrated that the binding of [^99m^Tc]Tc-(HE)_3_-E01 and [^123^I]I-(HE)_3_-E01-PIB to EGFR-expressing cell lines was EGFR-mediated and proportional to EGFR expression. The binding assay of [^123^I]I-(HE)_3_-E01 variant to the A-341 cell line with a high EGFR expression also showed specificity. However, the binding of [^123^I]I-(HE)_3_-E01 was much lower compared with the other two variants. We concluded that the tyrosine modification resulted in a substantial loss of binding capacity of [^123^I]I-(HE)_3_-E01, and this variant was excluded from further evaluation.

#### 2.3.2. Internalization

**Figure 3:**
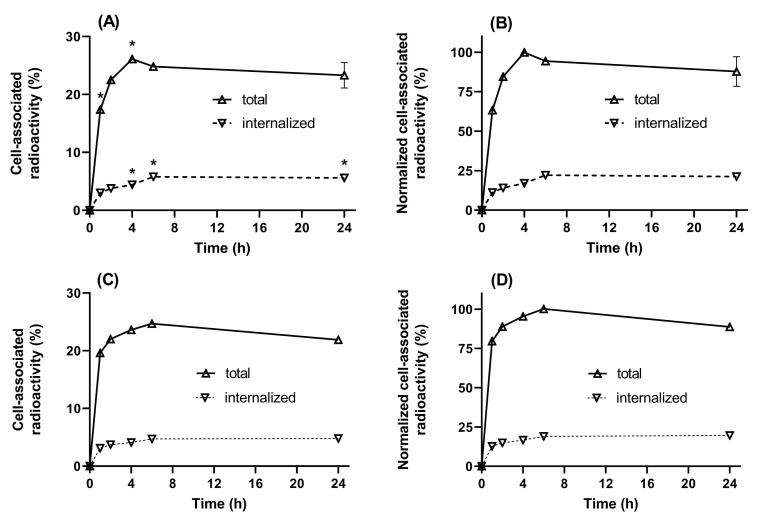
Cellular processing of (**A**,**B**) [^99m^Tc]Tc-(HE)_3_-E01 and (**C**,**D**) [^123^I]I-(HE)_3_-E01-PIB by A431 cells in vitro. Cell-associated activity was normalized to the maximum uptake (**B**,**D**). Data are presented as the mean of three samples ± standard deviation (SD). Error bars might not be seen because they are smaller than the symbols. To show significant differences (*p* < 0.05), data from internalization studies (between each point time for two labelled variants) were analyzed by an unpaired 2-tailed *t*-test. Asterisk (*) was used to show significant (*p* < 0.05) differences.

Data concerning the internalization of [^99m^Tc]Tc-(HE)_3_-E01 and [^123^I]I-(HE)_3_-E01-PIB by A-431 cells during continuous incubation are presented in Figure 3. The processing of bound [^99m^Tc]Tc-(HE)_3_-E01 and [^123^I]I-(HE)_3_-E01-PIB by A-431 cell line was studied by the acid wash method. The common feature of both [^99m^Tc]Tc-(HE)_3_-E01 and [^123^I]I-(HE)_3_-E01-PIB was rapid binding and relatively slow internalization by the A-431 cell line. The level of internalized activity reached a plateau at 6 h after the initiation of incubation. The internalized fraction of [^123^I]I-(HE)_3_-E01-PIB and [^99m^Tc]Tc-(HE)_3_-E01 at 24 h was 4.8 ± 0.2% and 5.7 ± 0.2%, respectively. There was a significant difference between the internalized fractions of [^123^I]I-(HE)_3_-E01-PIB and [^99m^Tc]Tc-(HE)_3_-E01 at 4, 6, and 24 h (*p* < 0.05). The total cellular activity for both variants exhibited a maximum at 4 to 6 h, followed by a subsequent slow decline. The total cell-bound activity of [^123^I]I-(HE)_3_-E01-PIB tended to be lower at 24 h compared to [^99m^Tc]Tc-(HE)_3_-E01 (*p* > 0.05 in unpaired *t*-test).

#### 2.3.3. Saturation Assay

**Figure 4:**
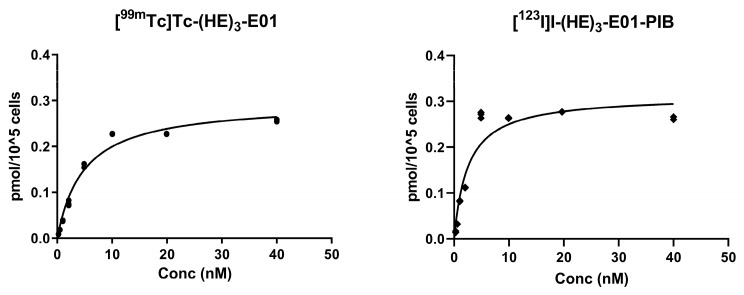
Determination of equilibrium dissociation constants (*K_D_*) of radiolabeled (HE)_3_-E01 variants by EGFR-expressing A-431 cells in vitro. The data are presented as measurements from three samples for each concentration. The dots and diamonds in the figure represent individual measured values.

This assay was performed to evaluate the equilibrium dissociation constant (K_D_), i.e., the affinity, of [^99m^Tc]Tc-(HE)_3_-E01 and [^123^I]I-(HE)_3_-E01-PIB binding to EGFR expressed by A431 cells. The saturation binding curve of radiolabeled (HE)_3_-E01 variants was observed (Figure 4), and the binding was significantly suppressed with a 50-fold molar excess of unlabeled (HE)_3_-E01 at each data point. The equilibrium dissociation constants of [^99m^Tc]Tc-(HE)_3_-E01 and [^123^I]I-(HE)_3_-E01-PIB were 4.9 ± 0.5 and 2.5 ± 0.5 nM, respectively, indicating similar affinities in the single-digit nanomolar range. The number of binding sites (*B*_max_) was determined to be 1.77–1.88 × 10^6^ receptor sites per cell.

### 2.4. In Vivo Studies

**Figure 5:**
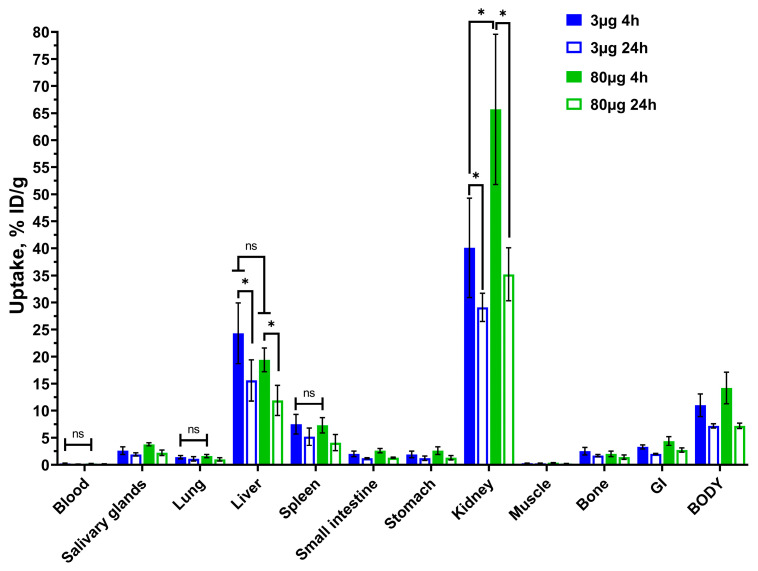
Biodistribution of [^99m^Tc]Tc-(HE)_3_-E01 after injection of 3 or 80 µg of the probe evaluated at 4 h and 24 h p.i. in CD1 mice. Data are presented as mean %ID/g ± SD for four mice. Data for the rest of the GI tract with content and the rest of the body are presented as %ID per whole sample. One-way ANOVA with Bonferroni’s multiple comparisons test was performed to find significant differences between time points and doses. An asterisk indicates that the differences between groups were significant (*p* ˂ 0.05), “ns” indicates that the differences between groups were not significant (*p* > 0.05).

Initially, the biodistribution of [^99m^Tc]Tc-(HE)_3_-E01 after injection of 3 or 80 µg of the probe per mouse at 4 h and 24 h post-injection (p.i.) in CD1 healthy mice was evaluated (Figure 5, Table S1). The [^99m^Tc]Tc-(HE)_3_-E01 demonstrated rapid blood clearance. Four hours p.i., the blood uptake for both doses of [^99m^Tc]Tc-(HE)_3_-E01 was no more than 0.30 ± 0.10% ID/g. On the contrary, the kidney uptake of the [^99m^Tc]Tc-(HE)_3_-E01 was high. The liver was another organ with prominent uptake (24.3 ± 5.6% and 19.4 ± 2.2% ID/g for doses of 3 µg and 80 µg, respectively, at four h post-injection). The radioactivity uptake in the gastrointestinal tract for both doses at four hours was low (no more than 4.40 ± 0.80% ID per whole sample, including content). There was no significant difference (*p* > 0.05) of uptake in blood, lung, liver, small intestine, stomach, bone, spleen, gastrointestinal tract, and the rest of the body between 3 µg and 80 µg of [^99m^Tc]Tc-(HE)_3_-E01 at four h, and even 24 h p.i. The absence of the blocking effect in normal organs (first and foremost in the liver) suggests that the uptake of radiolabeled E01 in these organs was not EGFR-specific.

**Figure 6:**
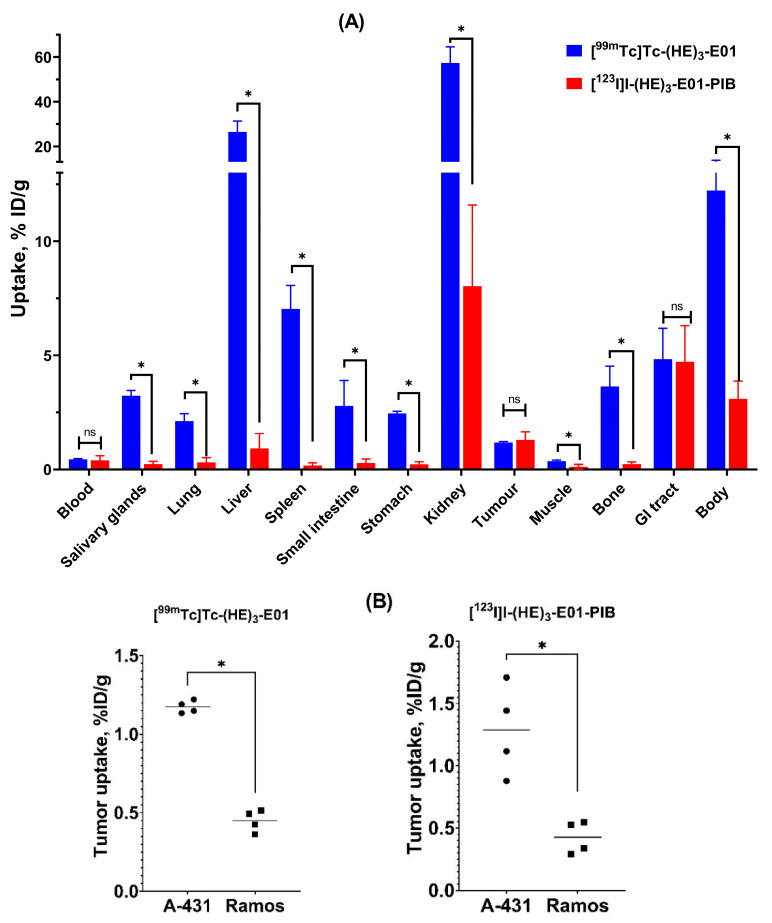
In vivo tumour targeting. (**A**). Comparative biodistribution of [^99m^Tc]Tc-(HE)_3_-E01 and [^123^I]I-(HE)_3_-E01-PIB in Nu/J mice bearing EGFR-expressing A-431 at 4 h p.i. (**B**). Comparison of the uptake of labeled DARPin (HE)_3_-E01 variants in EGFR-expressing A-431 and non-EGFR-expressing Ramos xenografts in Nu/J mice 4 h p.i. Data are presented as mean ± SD for four mice. Data for the rest of the GI tract with contents and the rest of the body are presented as %ID per whole sample. An asterisk indicates that the differences between groups were significant (*p* ˂ 0.05), “ns” indicates that the differences between groups were not significant (*p* > 0.05). The dots and squares in the figure are a graphic representation of individual result values.

Nu/J mice bearing EGFR-positive A-431 and EGFR-negative Ramos xenografts were used to evaluate the in vivo specificity of EGFR targeting by (HE)_3_-E01 labeled with [^99m^Tc]Tc-tricarbonyl and [^123^I]I-para-iodobenzoate (Figure 6 and Table S2). The accumulation of both variants was much higher in A-431 xenografts than in Ramos xenografts (*p* < 0.05, unpaired *t*-test), which demonstrated that the tumor uptake of both [^99m^Tc]Tc-(HE)_3_-E01 and [^123^I]I-(HE)_3_-E01-PIB was EGFR-specific (Figure 6B). There was no significant (*p* > 0.05, unpaired *t*-test) difference between tumor uptake of [^99m^Tc]Tc-(HE)_3_-E01 and [^123^I]I-(HE)_3_-E01-PIB in A-431 xenografts (1.17 ± 0.04 and 1.29 ± 0.36%ID/g, respectively) (Figure 6A). No differences in uptake of both variants in normal organs between A-431 and Ramos groups were detected (*p* > 0.05, unpaired *t*-test), except for small but significant differences in gastrointestinal tract uptake for [^123^I]I-(HE)_3_-E01-PIB (*p* < 0.05, unpaired *t*-test) (Table S2).

There were notable disparities in the distribution of activity in the normal organs and tissues between radioiodinated and ^99m^Tc-labeled E01 (Figure 6A and Table S2). The [^123^I]I-(HE)_3_-E01-PIB uptake in kidney (8.0 ± 3.6%ID/g) was significantly (seven-fold) lower than the [^99m^Tc]Tc-(HE)_3_-E01 uptake (57 ± 7%ID/g). Hepatic uptake of [^123^I]I-PIB-(HE)_3_-E01 was *28*-fold lower than that of [^99m^Tc]Tc-(HE)_3_-E01 (0.9 ± 0.7 and 26.5 ± 4.8%ID/g, respectively). The [^123^I]I-(HE)_3_-E01-PIB uptake value in the tumor was higher than that in normal organs and tissues, except the kidney. Overall, the [^123^I]I-(HE)_3_-E01-PIB had significantly (*p* < 0.05, unpaired *t*-test) lower retention of activity in normal organs and tissues than that of [^99m^Tc]Tc-(HE)_3_-E01, including in salivary glands, lung, spleen, small intestine, stomach, muscle, and bone.

**Figure 7:**
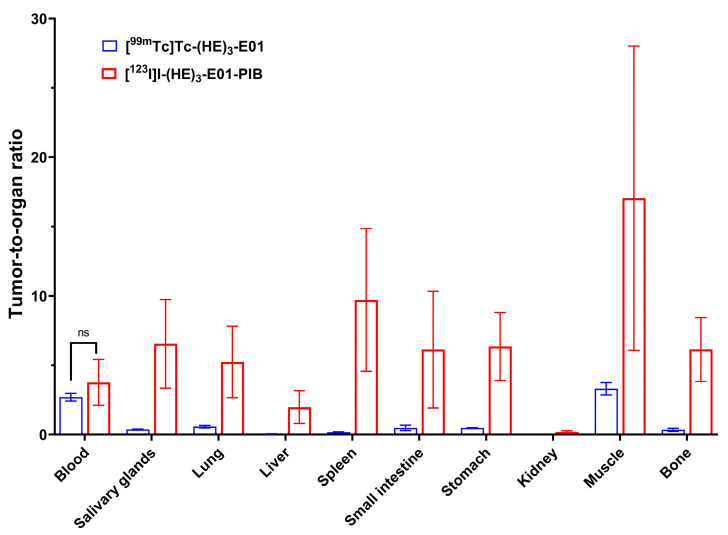
Tumor-to-organ ratios of [^99m^Tc]Tc-(HE)_3_-E01 and [^123^I]I-(HE)_3_-E01-PIB at 4 h post-injection (p.i.) in Nu/j mice bearing A-431 xenografts. Data are presented as mean ± SD for four mice. “ns” indicates that the differences between groups were not significant (*p* > 0.05).

The tumor-to-organ ratios for [^99m^Tc]Tc-(HE)_3_-E01 and [^123^I]I-(HE)_3_-E01-PIB at 4 h post injection in Nu/j mice bearing A-431 xenografts are shown in Figure 7. The lower [^123^I]I-(HE)_3_-E01-PIB uptake in all normal organs and tissues resulted in significantly higher tumor-to-organ ratios compared with [^99m^Tc]Tc-(HE)_3_-E01. At 4 h p.i., [^123^I]I-(HE)_3_-E01-PIB provided *15*-fold higher tumor-to-salivary glands, *eight*-fold higher tumor-to-lung, *32*-fold higher tumor-to-liver, *44*-fold higher tumor-to-spleen, *10*-fold higher tumor-to-small intestine, *12*-fold higher tumor-to-stomach, *10*-fold higher tumor-to-kidney, *three*-fold higher tumor-to-muscle, and *17*-fold higher tumor-to-bone ratio compared to that of [^99m^Tc]Tc-(HE)_3_-E01 (data is presented in Table S3 in Supplementary Information).

**Figure 8:**
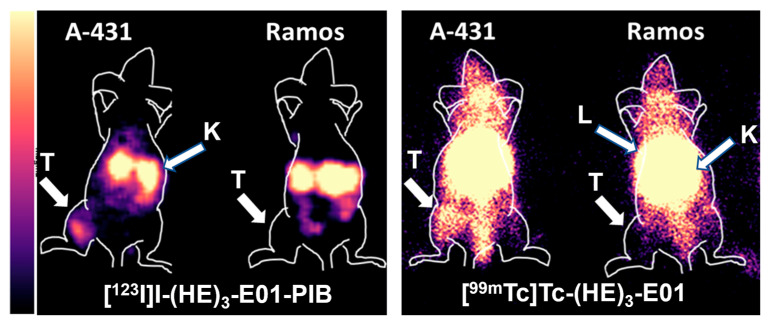
Gamma-camera imaging of EGFR expression in Nu/J mice bearing A-431 and Ramos xenografts using [^99m^Tc]Tc-(HE)_3_-E01 and [^123^I]I-(HE)_3_-E01-PIB 4 h post-injection. Three µg (4 MBq) for [^99m^Tc]Tc-(HE)_3_-E01 and 3 µg (1.7 MBq) for and [^123^I]I-(HE)_3_-E01-PIB. Contours were derived from a digital photograph and superimposed over the images to facilitate interpretation. L—liver, K—kidneys, T—tumor. Settings of a linear magma scale were adjusted on each image to provide visualization of EGFR-positive A-431 xenografts.

Gamma-camera imaging using [^99m^Tc]Tc-(HE)_3_-E01 and [^123^I]I-(HE)_3_-E01-PIB in Nu/J mice bearing A-431 and Ramos-xenografts at 4 h p.i. confirmed the results of the biodistribution studies (Figure 8). The use of [^123^I]I-PIB-(HE)_3_-E01 enabled clear visualization of A-431 xenografts. The tumor-to-kidney, tumor-to-liver, and tumor-to-contralateral site ratios were 0.54, 0.76, and 3.9, respectively. Imaging using the technetium-labeled variant appears to be poor, with little or no contrast between the accumulation of activity in the tumor and healthy organs and tissues. The tumor-to-kidney, tumor-to-liver, and tumor-to-contralateral site ratios were 0.1, 0.08, and 2.2, respectively.

## 3. Discussion

(HE)_3_-E01 DARPin was site-specifically labeled in a high yield with technetium-99m tricarbonyl using a (HE)_3_ tag placed at the N-terminus (Table 1). This label is residualizing, i.e., it is retained inside the cell after internalization. Two variants of DARPin (HE)_3_-E01 with non-residualizing labels were obtained via direct and indirect electrophilic radioiodination. By direct electrophilic radioiodination, ^123^I was bound in a high yield (Table 1) to one of four tyrosines in E01. The indirect electrophilic radioiodination was performed through conjugation of N-succinimidyl-*para*-[^123^I]I-iodobenzoate to one of the eight lysine amino groups in (HE)_3_-E01 or its N-terminal amino group. In this case, the radiochemical yield was noticeably lower compared to two other methods (Table 1), but size-exclusion chromatography enabled obtaining a high radiochemical purity of [^123^I]I-(HE)_3_-E01-PIB.

All radiolabeled (HE)_3_-E01 variants retained the capacity of specific binding to EGFR-expressing cells. This was shown by a significant reduction in their binding to EGFR-expressing cells in the presence of a large excess of both unlabeled (HE)_3_-E01 and anti-EGFR antibody cetuximab (Figure 2). Like cetuximab, E01 binds to an epitope located near the C-terminal end of EGFR domain III [42]. The competition by cetuximab demonstrates EGFR specificity beyond any reasonable doubt. Furthermore, the specificity test results demonstrated that the binding of radiolabeled [^99m^Tc]Tc-(HE)_3_-E01 and [^123^I]I-(HE)_3_-E01-PIB EGFR-expressing cell lines was proportional to the receptor expression levels. However, the binding magnitude of [^123^I]I-(HE)_3_-E01 was clearly inferior to the magnitude of binding of both [^99m^Tc]Tc-(HE)_3_-E01 and [^123^I]I-(HE)_3_-E01-PIB to the EGFR-expressing A-431 cells (Figure 2). Direct iodination using Chloramine-T is a robust and straightforward method that can be applied for labeling using clinically relevant iodine radioisotopes [50]. However, modification of tyrosines might be detrimental for binding. In this study, the amount of carrier was selected to provide the incorporation of a single iodine atom per two molecules of DARPin. This should not result in a more than tenfold reduction in binding (Figure 2) compared to other methods. However, it is important to remember that the use of Chloramine-T also causes the chlorination of tyrosines [50], and the extent of the chlorination is difficult to control. This might be the most probable reason for the loss of [^123^I]I-(HE)_3_-E01 specific binding to EGFR-expressing cells. Based on this consideration, [^123^I]I-(HE)_3_-E01 was not continued in further characterizations and in vivo evaluations.

The cellular processing of [^99m^Tc]Tc-(HE)_3_-E01 and [^123^I]I-(HE)_3_-E01-PIB revealed a relatively low percentage of internalized fractions, which is typical for DARPin-based binders to other targets [46,48]. The internalization pattern of both radiolabeled (HE)_3_-E01 variants by A-431 was similar. The internalized fraction was not lower for DARPin labeled with the non-residualizing iodine-123 label in comparison to the residualizing tricarbonyl technetium-99m label. This finding implies that the residualizing properties of the label may not be indispensable for effective activity retention by malignant cells. Conversely, the preservation of sufficient affinity was deemed to be of greater significance [46]. Both variants demonstrated affinities in the low nanomolar range in the saturation binding assay (Figure 4). We considered the combination of specific binding, the slow internalization, and high affinity of [^99m^Tc]Tc-(HE)_3_-E01 and [^123^I]I-(HE)_3_-E01-PIB as a good rationale to proceed with in vivo experiments.

Biodistribution study of [^99m^Tc]Tc-(HE)_3_-E01 with different doses, which were evaluated in CD1 mice at 4 h and 24 h p.i. (Figure 5) indicated that [^99m^Tc]Tc-(HE)_3_-E01 had a low accumulation of activity in blood, small intestine, stomach, muscle, and bone. The renal and hepatic uptake of [^99m^Tc]Tc-(HE)_3_-E01 was high. The high kidney uptake suggested that [^99m^Tc]Tc-(HE)_3_-E01 underwent a rapid glomerular filtration followed by renal reabsorption, which is typical for DARPin molecules. Although the liver was the other organ with prominent uptake, the low radioactivity uptake in the gastrointestinal tract suggested that hepatobiliary excretion played a minor role in the clearance of [^99m^Tc]Tc-(HE)_3_-E01. There was no significant difference in uptake in the liver between 3 µg and 80 µg of [^99m^Tc]Tc-(HE)_3_-E01. Two different mechanisms might mediate the hepatic uptake of anti-EGFR DARPin molecules. One mechanism is EGFR-dependent. The second mechanism is independent of EGFR binding but depends on the physicochemical properties of a protein and a chelator/prosthetic group-radionuclide combination, which also significantly influences hepatic uptake [22]. Absence of saturation of the hepatic uptake after co-injection of a large excess of (HE)_3_-E01 suggests that E01 does not bind to murine EGFR. It has to be noted that the binding to murine EGFR was not evaluated in this study. However, earlier studies with affibodies having equal affinity to human and murine EGFR [16,45] demonstrated clear saturation of the uptake in the liver and significant reduction in the hepatic uptake when equivalent masses were injected in mice. This has not been observed in this study. Thus, the EGF-independent mechanism is decisive in the hepatic uptake of [^99m^Tc]Tc-(HE)_3_-E01. The difference between uptake of [^99m^Tc]Tc-(HE)_3_-E01 in liver and kidneys at 4 h and 24 h p.i. was minor, which indicates that the tracer was internalized by these organs, but the radionuclide was retained due to the residualizing properties of the ^99m^Tc label.

[^99m^Tc]Tc-(HE)_3_-E01 and [^123^I]I-(HE)_3_-E01-PIB were injected into A-431 and Ramos tumor-bearing mice to compare their biodistribution and tumor targeting capacity. The uptake of both [^99m^Tc]Tc-(HE)_3_-E01 and [^123^I]I-(HE)_3_-E01-PIB was significantly (*p* < 0.05) higher in A-431 compared to Ramos xenografts (Figure 6). This clearly indicated that the tumor accumulation of both tracers was EGFR-mediated. There was no significant difference between the uptake of [^99m^Tc]Tc-(HE)_3_-E01 and [^123^I]I-(HE)_3_-E01-PIB in EGFR-positive tumors. The biodistribution of both radiolabeled (HE)_3_-E01 variants was typical for DARPins specific to other targets, with rapid clearance from the blood (remaining less than 0.5% ID/g at 4 h p.i.) via the kidneys, followed by renal reabsorption, and retention of activity in the kidneys. The low gastrointestinal tract accumulation of activity suggests that the renal excretion pathway was predominant for both radiolabeled (HE)_3_-E01 DARPins. However, the renal uptake of [^99m^Tc]Tc-(HE)_3_-E01 showed seven-fold higher accumulation activity than that of [^123^I]I-(HE)_3_-E01-PIB. The uptake of [^99m^Tc]Tc-(HE)_3_-E01 in the majority of normal organs was also significantly higher than the uptake of [^123^I]I-(HE)_3_-E01-PIB. This biodistribution pattern is concordant with the pattern for other DARPins (targeting HER2 or EpCAM) having lower normal tissue uptake with non-residualizing labels than with residualizing ones [46,48,51]. This shows that the radiolabeled (HE)_3_-E01 is internalized after nonspecific binding to normal tissues. In this case, the non-residualizing radioiodine leaks from cells in excretory organs and is excreted via the kidneys, while radiometals (including ^99m^Tc) are retained intracellularly. However, the slow internalization of DARPins by malignant cells facilitates a good retention of radiolabeled DARPins in tumors, where they remain bound to their targets expressed on cellular membranes. Importantly, the liver uptake of [^99m^Tc]Tc-(HE)_3_-E01 was higher than the tumor uptake. This is undesirable because the liver is the primary site for metastasis in numerous cancers [52]. Previous investigation with other engineered scaffold proteins (ESPs), affibody molecules [53], DARPin G3 [47], and DARPin Ec1 [46], demonstrated that substituting the H_6_ tag with the more hydrophilic negatively charged (HE)_3_-tag resulted in decreased liver uptake when labeled with ^99m^Tc(CO)_3_. However, the application of this tag did not reduce the hepatic absorption of ADAPT ESP compounds, which were labeled identically using ^99m^Tc(CO)_3_ [54]. Thus, it is impossible to generalize that the utilization of the (HE)_3_ tag would result in the same impact on biodistribution for all ESPs. The use of a hydrophilic and negatively charged (HE)_3_ tag at the N-terminus of DARPin molecules in this study did not enable obtaining a low hepatic uptake [^99m^Tc]Tc-(HE)_3_-E01. This goal was achieved by the use of [^123^I]I-(HE)_3_-E01-PIB. An important aspect of the use of indirect radioiodination is the rapid excretion of radiometabolites, without their uptake in organs expressing the Na/I symporter. This contributes to a better imaging contrast. The shortcoming of the use of indirect iodination is its lower yield compared with the direct method. The low radiochemical yields are typical for indirect radioiodinations utilizing N-succinimidyl esters. This occurs because there are two competing reactions during the conjugation step: aminolysis of the N-succinimide ester, resulting in the covalent coupling of a radiolabeled prosthetic group to the primary amines of a protein, and hydrolysis, resulting in the formation of free radiolabeled acid. However, low yields are accepted in clinical translation when a label provides a favorable biodistribution. For example, a Phase I clinical trial of anti-HER2 VHH1 labeled using [^131^I]I-N-succinimidyl 4-guanidinomethyl-3-iodobenzoate radiolabeling for therapy was performed, although the radiochemical yield was 6.1 ± 1.7% [55].This study demonstrated that the properties of a radioactive label (i.e., radionuclide and a chelator/prosthetic group for its attachment to a targeting protein) influence the biodistribution of (HE)_3_-E01 DARPin. The results of the biodistribution experiments demonstrated the advantages of using the non-residualizing ^123^I-PIB label for slowly internalizing high-affinity imaging radiotracers. The [^123^I]I-(HE)_3_-E01-PIB can be proposed as a candidate for further development of a radiotracer for detecting EGFR expression levels in malignant tumors, providing important prognostic and predictive information that can influence the stratification of cancer patients for EGFR-targeting therapies. Comparison with antibody-based radiolabeled EGFR imaging probes, which were tested using the same A431 xenograft model, shows that the tumor uptake of [^123^I]I-(HE)_3_-E01-PIB at 4 h after injection (1.29 ± 0.36%ID/g) was lower than the peak uptake of ^89^Zr-labeled cetuximab (~4%ID/g at 96 h after injection [17] or ^86^Y-labeled panitumumab (22.74 ± 1.7%ID/g) [56]. However, to tumor-to-blood ratio for [^123^I]I-(HE)_3_-E01-PIB (3.8 ± 1.7%ID/g) is comparable to corresponding values for these tracers (~1.5 and ~3, for [^89^Zr]Zr-DFO-cetuximab and [^86^Y]Y-CHX-A’’-DTPA-panitumumab, respectively). Several variants of EGFR-targeting affibodies [22] demonstrated higher tumor uptake (range between 2.7 and 8.6%ID/g at 3 h after injection), and tumor-to-blood ratios were also higher for these tracers (range between 7 and 12). It is important to keep in mind that these affibody variants were developed after several years of intensive research, while this paper is the very first report describing the evaluation of radiolabeled E01 in vivo.

## 4. Materials and Methods

### 4.1. General Materials and Instruments

The molecular weight of the DARPin (HE)_3_-E01 was determined by an Ultimate 3000 Nano LC System (Thermo Fisher Scientific, Waltham, MA, USA), which was coupled to the Orbitrap Tribrid Lumos mass spectrometer (Thermo Fisher Scientific, Waltham, MA, USA) with a nanoelectrospray source (Thermo Fisher Scientific, Waltham, MA, USA). The purified protein concentration was determined using a NanoDrop OneC (Thermo Scientific, Waltham, MA, USA). The ^123^I was obtained as [^123^I]NaI from the Tomsk Polytechnic University R-7M cyclotron facility. Technetium-99m was obtained as pertechnetate by elution of a commercial ^99^Mo/^99m^Tc generator, GT-4K (FSUE “Karpov Institute of Physical Chemistry,” Obninsk, Russia). The CRS (Center for Radiopharmaceutical Sciences) kits for preparing tricarbonyl technetium were purchased from the Center for Radiopharmaceutical Sciences (PSI, Villigen, Switzerland). Instant thin-layer chromatography (iTLC) was performed using iTLC silica gel strips (Agilent Technologies, Inc., Folsom, CA, USA) and quantified by the miniGITA Single iTLC scanner (Elysia Raytest, Straubenhardt, Germany). Purification of radiolabeled proteins was performed using NAP-5 size-exclusion columns (Cytiva, Uppsala, Sweden). The radioactivity in vitro and in vivo test samples was measured using a thallium-activated sodium iodide (NaI(Tl)) detector and an automated gamma counter, Wizard 1480 (PerkinElmer, Waltham, MA, USA). The radioactivity of the injection solution for the in vivo tests was measured using an ionization chamber with a dose calibrator, RIS-1A, Amplituda, Saint Petersburg, Russia. Whole-body gamma-camera imaging of mice was performed using a Siemens E.Cam 180 scanner equipped with a high-resolution, low-energy collimator (Siemens, Erlangen, Germany).

The results are displayed as the mean value with a standard deviation of three for cell studies and four for animal studies. GraphPad Prism (version 9.5.0 for Windows; GraphPad Software, La Jolla, CA, USA) was used to generate plots and perform statistical analysis. Two-tailed t-tests were used to determine any significant difference between two different groups.

### 4.2. Cell Lines

The human cancer cell lines with different levels of EGFR expression, A-431 (epidermoid carcinoma), DU-145 (prostate adenocarcinoma), and U-87 MG (glioblastoma), as well as PC-3 (prostate carcinoma) with very low expression of EGFR, were purchased from the American Type Culture Collection (ATCC, Manassas, VA, USA). The cells were cultured in Roswell Park Memorial Institute (RPMI) medium supplemented with 10% fetal bovine serum (FBS), two mM L-glutamine, 100 IU/mL penicillin, and 100 µg/mL streptomycin in a humidified incubator with 5% CO_2_ at 37 °C, unless indicated otherwise. The cells were detached by trypsin-ethylenediaminetetraacetic acid (EDTA) solution (25200056, Thermo Fisher Scientific, Waltham, MA, USA). The in vitro experiments were performed using 35 mm Petri dishes (Nunclon Delta Surface, ThermoFisher Scientific, Roskilde, Denmark).

### 4.3. Protein Production and Characterization

The nucleotide sequence of DARPin E01 gene was deduced from the amino acid sequence of DARPin E01 taken from [57]. DARPin (HE)_3_-E01 was produced in the *E. coli* strain and purified using the methodology described earlier [48]. DARPin (HE)_3_-E01 contains glutamate-histidine-glutamate-histidine-glutamate ((HE)_3_) tag placed at the *N*-terminus. Its amino acid sequence is as follows: MRGSHEHEHEGSDLGKKLLEAARAGQDDEVRILMANGADVNADDTWGWTPLHLAAYQGHLEIVEVLLKNGADVNAYDYIGWTPLHLAADGHLEIVEVLLKNGADVNASDYIGDTPLHLAAHNGHLEIVEVLLKHGADVNAQDKFGKTAFDISIDNGNEDLAEILQKLN. The purified DARPin (HE)_3_-E01 concentration was determined using a NanoDrop OneC (Thermo Fisher Scientific, Wilmington, DE, USA) using ε280 = 22,460 M^−1^ cm^−1^.

The molecular weight of the DARPin (HE)_3_-E01 was determined by liquid chromatography-electrospray ionization mass spectrometry (LC-ESI/MS). Protein sample (200 ng) was loaded to a home-made trap column 50 × 0.1 mm, packed with Reprosil PUR 200 C18-AQ, 5 µm (Dr. Maisch), in the loading mobile phase (2% acetonitrile (ACN), 98% H_2_O, 0.1% TFA) at 4.2 µL/min flow and separated at RT in a home-packed [58] fused-silica column 300 × 0.1 mm packed with Reprosil PUR 200 C18-AQ, 1.9 µm (Dr. Maisch) into an emitter prepared with P2000 Laser Puller (Sutter, Novato, CA, USA). Reverse-phase chromatography was performed with an Ultimate 3000 Nano LC System (Thermo Fisher Scientific, Sunnyvale, CA, USA), which was coupled to the Orbitrap Tribrid Lumos mass spectrometer (Thermo Fisher Scientific) via a nanoelectrospray source (Thermo Fisher Scientific). Water containing 0.1% (*v*/*v*) FA was used as mobile phase A and ACN containing 0.1% FA (*v*/*v*), 20% (*v*/*v*) H2O as mobile phase B. Protein was eluted from the trap column with a linear gradient: 15% B for 3 min; 15–70% B during 16 min, 70–90% B during 0.1 min, 90% B for 4 min, 90–15% B during 0.1 min at a flow rate of 500 nL/min. MS parameters were as follows: 120K resolution, 500–2000 scan range, max injection time—auto, AGC target—standard.

### 4.4. Synthesis of N-Succinimidyl-p-(Trimethylstannyl)Benzoate

N-succinimidyl-*p*-(trimethylstannyl)benzoate was synthesized using a modification of a previously published method [49]. Firstly, *p-*(trimethylstannyl)benzoic acid was prepared (Figure S5). One mL of hexamethyldistannane ((CH_3_)_6_Sn_2_, 4.8 mmol) and 20 mg bis-(triphenylphosphine)-palladium(II)-dichloride ((P(Ph_3_))_2_Pd(II)Cl_2_, 0.03 mmol) were added to a Schlenk flask containing a solution of *p*-iodobenzoic acid (0.5 g, 2 mmol) in 10 mL of degassed 1,4-dioxane. The solution was stirred at 60 °C for 4 h in the argon atmosphere. After evaporation, the crude mixture was applied to a chromatography column and eluted with an EtOAc:hexane gradient (100% hexane to 60% hexane: 40% EtOAc) to yield solid *p*-(trimethylstannyl)benzoate. The ^1^H NMR spectra of p-(trimethylstannyl)benzoic acid are presented in Figure S5 (in Supplementary Information). ^1^H NMR (CDCl_3_, 400 MHz) δ 8.05 (2H, dd, *J* = 8.0 Hz), 7.63 (2H, dd, *J* = 8.0 Hz), 0.33 (9H, s).

Secondly, 388.1 mg of *p*-(trimethylstannyl)benzoic acid (1.36 mmol), 313.24 mg of 1-ethyl-3-(3-dimethylaminopropyl)carbodiimide hydrochloride (1.63 mmol), and 188.2 mg N-hydroxysuccinimide (1.63 mmol) were added to a Schlenk Flask containing 5 mL of dichloromethane (Figure S5). The mixture was stirred for 8 h at room temperature in an argon atmosphere and then evaporated to dryness. After that, the raw product was applied to a chromatography column and eluted with an EtOAc: hexane gradient (20% EtOAc: 80% hexane to 50% EtOAc: 50% hexane), yielding a white crystalline solid of N-succinimidyl-p-(trimethylstannyl)benzoate. The ^1^H NMR and ^13^C NMR spectra of N-succinimidyl-p-(trimethylstannyl)benzoate are presented in Figures S6 and S7 (in Supplementary Information). ^1^H NMR (CDCl_3_, 400 MHz) δ 8.03 (2H, dd, *J* = 8.2 Hz), 7.63 (2H, dd, *J* = 8.2 Hz), 2.89 (4H, s), 0.33 (9H, s). ^13^C NMR (101 MHz, CDCl_3_) δ 169.29, 162.28, 153.11, 136.20, 129.23, 124.65, 25.71, −9.45.

### 4.5. Radiolabeling

DARPin E01 was site-specifically labeled with technetium tricarbonyl [^99m^Tc][Tc(CO)_3_]^+^ using a N-terminal (HE)_3_-tag as described previously [59] with a slight modification. The eluate containing [^99m^Tc]TcO_4_^−^ in 1000 µL of 0.9% NaCl (2–4 GBq) was introduced into a vial containing a CRS kit. Subsequently, the mixture was incubated at 100 °C for 30 min to produce the [^99m^Tc]Tc(CO)_3_^+^ precursor. Afterward, the solution was allowed to cool to room temperature for 10 min. Then, 100 µL [^99m^Tc]Tc(CO)_3_^+^ from the CRS reaction mixture (100–200 MBq) was added to DARPin E01-(HE)_3_ solution (50 µg, 50 µL of 4.96 mg/mL in PBS), and 100 µL 0.1 M HCl was added to adjust pH to 7.5–8. The mixture was incubated at 60 °C for 60 min. After incubation, radiolabeled DARPins were isolated using size-exclusion chromatography on disposable NAP-5 columns, pre-equilibrated and eluted with PBS. The radiochemical yield and purity of [^99m^Tc]Tc-(HE)_3_-E01 were determined using radio-iTLC analysis in a PBS system.

DARPin (HE)_3_-E01 was directly labeled with iodine-123 using the chloramine-T method, which was adapted from a previously reported [49]. To [^123^I]NaI solution (50 µL, 25–30 MBq), 0.1 M HCl solution (5 µL) was added. Then, DARPin (HE)_3_-E01 (2.7 nmol, 50 µg, 10 µL of 4.96 mg/mL in 0.02 M phosphate buffer) and NaI solution (1.4 nmol, 0.2 µg, 2 µL of 0.1 mg/mL) were added to the reaction mixture, and the mixture was vortexed. Five minutes later, chloramine-T (40 µg, 10 µL of 4 mg/mL in PBS) was added. The reaction was continued at room temperature for two minutes. To stop the reaction, sodium metabisulfite solution (80 µg, 10 µL, 8 mg/mL in water) was added. The radiolabeled DARPin (HE)_3_-E01 was purified using size-exclusion chromatography on disposable NAP-5 columns, pre-equilibrated and eluted with PBS. The radiochemical yield and purity of [^123^I]I-(HE)_3_-E01 were determined using radio-iTLC analysis in 4:1 acetone/water.

Indirect labeling of DARPin (HE)_3_-E01 was performed in two steps similar to the methodology previously described by Vorobyeva et al. [60]. First, 0.1% CH_3_COOH solution (10–30 µL) was added to [^123^I][NaI solution (100–300 µL, 50–200 MBq). Then, N-succinimidyl-*p*-(trimethylstannyl)benzoate (8 nmol, 4 µg, 4 µL of 1 mg/mL previously dissolved in CH_3_OH/CH_3_COOH; 95/5 (*v*/*v*) was added to the ^123^I solution. Iodination was started by adding chloramine-T (40 µg, 10 µL, 4 mg/mL in water). The reaction was performed at room temperature for five minutes. To stop the reaction, sodium metabisulfite solution (60 µg, 10 µL, 6 mg/mL in water) was added to the reaction mixture. The radiochemical yield of [^123^I]I-PIB was measured using radio-iTLC with ethyl acetate as a mobile phase. Then, DARPin (HE)_3_-E01 (10.94 nmoles, 200 µg, 40 µL of 4.96 mg/mL in 0.02 M phosphate buffer) and 120 µL of 0.07 M borate buffer (pH 9.3) were added to the reaction mixture. After 30 min of incubation at room temperature, the radiolabeled conjugate was purified using size-exclusion chromatography on disposable NAP-5 columns, which were pre-equilibrated and eluted with PBS. The radiochemical yield and purity of [^123^I]I-(HE)_3_-E01-PIB were determined using radio-iTLC analysis in a 4:1 acetone/water system.

### 4.6. In Vitro Studies

The evaluation of the binding specificity of radiolabeled E01 to EGFR-expressing cells was performed as described previously [16] with slight modification. Four human cancer cell lines with different levels of EGFR expression, A-431 (~1.2 × 10^6^ receptors/cell) [61], DU-145 (~2 × 10^5^ receptors/cell) [62], and U-87 MG (~1.0 × 10^5^ receptors/cell) [58], as well as PC-3 (~3 × 10^4^ receptors/cell) [61], were used to determine the binding specificity of both radiolabeled DARPins (HE)_3_-E01. The cells (density of 10^6^ cells/well) were seeded in 6-well plates one day before the experiment. Two sets (six wells) of dishes were used for each cell line. The experiment was performed in triplicate. A 100-fold molar excess of unlabeled DARPin (HE)_3_-E01 or cetuximab was added to saturate EGFRs in one set of dishes (three dishes for each cell line). An equal volume of complete media was added to the other three dishes (for each cell line). After 30 min, a solution of radiolabeled DARPins (HE)_3_-E01 was added to each dish to reach a final concentration of 5 nM (app. 30–40 kBq per dish). After incubation for 1 h at 37 °C, the medium was collected, and the cells were washed with fresh medium (1 mL), which was then collected into the same tubes. Then, the cells were detached by treating them with 500 µL of trypsin for 10 min at 37 °C, and then collected. The cell-associated radioactivity in the fractions containing medium or cells was measured using a gamma counter and displayed as a percentage of cell-associated activity.

The cellular processing of radiolabeled [^99m^Tc]Tc-(HE)_3_-E01 and [[^123^I]I-(HE)_3_-E01-PIB by A-431 cells during continuous incubation was studied by an acid-wash method, which was validated earlier [63]. [^99m^Tc]Tc-(HE)_3_-E01 or [^123^I]I-(HE)_3_-E01-PIB (5 nM) were added to cells (ca. 10^6^ cells per dish, 3 dishes per time point), which were incubated at 37 °C for 1, 2, 4, 6, and 24 h. The supernatant was collected at the predetermined time intervals. Following that, the cells were rinsed once with PBS (1 mL) and subjected to a 5 min treatment with 0.2 M glycine buffer containing 4 M urea, pH 2.0 (1 mL), on ice in order to collect the membrane-bound fraction. Then, the cell debris containing the internalized conjugates was detached by treatment with 1 M NaOH for 30 min at 37 °C. The activity in every fraction was measured using a gamma spectrometer.

The equilibrium dissociation constant (*K_D_*) of [^99m^Tc]Tc-(HE)_3_-E01 or [^123^I]I-(HE)_3_-E01-PIB to living A-431 cells was determined using a saturation assay [61]. Cells were seeded in cell culture dishes (approximately 10^6^ cells per dish) one day before the experiments. Four dishes (3 containing cells with non-blocked and one with blocked EGFR) were used for each concentration. A total of three dishes were used to investigate specific binding, while a single cell culture dish was used to assess nonspecific binding by receptor blocking with a 50-fold molar excess of unlabeled (HE)_3_-E01. Eight radiolabeled protein concentrations (ranging from 0.22 to 40 nM) were used. To control dishes, medium (500 µL) containing unlabeled protein was added. To other dishes, a pure medium with serum (at the same volume) was added, and the cells were incubated in a humidified incubator (5% CO_2_, 37 °C) for 30 min. Then, the required concentration of the radiolabeled DARPin (HE)_3_-E01 was added to each set of cells (500 µL per dish), and the cells were incubated at 4 °C for 4 h. Thereafter, the medium with the radioactive solution was collected from each dish, followed by rinsing with PBS (5 times). Then, a trypsin–EDTA solution (500 µL per dish) was added, and the cells were further incubated for 10 min. Following the collection of all cells, 1 mL of medium was added to each dish. Afterward, 500 µL of the sample volume was collected into the cell counter, and the remaining 1 mL of the sample was measured for radioactivity. The radioactivity of the cells and the labeled protein standards was measured using an automated gamma counter. The *K_D_* was determined by a nonlinear regression analysis using GraphPad Prism software.

### 4.7. Animal Studies

Animal studies were planned and performed in accordance with the guidelines of the Russian Federation for the care and use of animals. The described procedures were approved by the Ethics Committee of Siberian State Medical University, Tomsk, Russia (protocol code 2, 20220927).

To study the characteristic of the radiolabeled DARPin (HE)_3_-E01 distribution in normal mice, and evaluate if the uptake in the liver is saturable, the biodistribution of [^99m^Tc]Tc-(HE)_3_-E01 with different doses was evaluated in CD1 mice at 4 h and 24 h p.i. [^123^I]I-(HE)_3_-E01-PIB was not used in this preliminary experiment because the release of radiometabolites of the non-residualizing radioiodine label from the liver, kidneys, and other organs would provide the data reflecting not only the distribution of the E01, but also its metabolism and release of radiometabolites. Briefly, 16 female CD1 mice (6 weeks of age) divided into 4 groups were intravenously (tail vein) injected with 3 µg (molar activities for 4 h and 24 h time points: 0.37 MBq/nmol and 3.9 MBq/nmol, respectively) and 80 µg (molar activities for 4 h and 24 h time points: 0.014 MBq/nmol and 0.146 MBq/nmol, respectively) of [^99m^Tc]Tc-(HE)_3_-E01 in 100 µL PBS with 1% BSA. At the time of the experiment, the average weights of the mice were 25.00 ± 0.82 g. Before dissection, mice were anesthetized by cervical dislocation. The organs and tissues were collected and weighed, and the activity was measured using an automated gamma spectrometer. The activity uptake was calculated as the percentage of the injected dose per gram of sample (% ID/g).

To evaluate the targeting characteristics of radiolabeled DARPins (HE)_3_-E01, the biodistribution study of [^99m^Tc]Tc-(HE)_3_-E01 and [^123^I]I-(HE)_3_-E01-PIB was performed in Nu/j mice bearing A-431 xenografts. For specificity control, Nu/j mice bearing Ramos xenografts were also implemented. To establish A-431 xenografts, 10^7^ cells in 100 µL media were implanted subcutaneously in female Nu/j mice, or the same amount of Ramos xenografts [64]. At the time of the experiment (2 weeks after implantation), the average weights of the animals were 25.25 ± 2.71 g in the A-431 group and 26.13 ± 1.74 g in the Ramos group. The average tumor weights measured after dissection were 0.54 ± 0.15 g for A-431 for A-431 and 0.07 ± 0.05 g for Ramos. Groups of four animals were used per data point. Two groups of mice were intravenously (via the tail vein) injected with three µg of [^99m^Tc]Tc-(HE)_3_-E01 (60 kBq, 100 µL in PBS with 1% BSA). Another two groups of mice were intravenously (via the tail vein) injected with three µg of [^123^I]I-(HE)_3_-E01-PIB (0.37 MBq/nmol, 40 kBq, 100 µL in PBS with 1% BSA). The biodistribution of radiolabeled DARPins (HE)_3_-E01 was measured four hours post-injection (p.i.). Before dissection, mice were anesthetized by cervical dislocation. Blood, organs, and carcasses were collected and weighed, and the activity was measured using an automated gamma spectrometer. The activity uptake was calculated as the percentage of the injected dose per gram of sample (% ID/g).

Whole-body gamma-camera imaging was performed using a Siemens E.CAM 180 scanner equipped with a high-resolution, low-energy collimator (Siemens, Germany). The Nu/j mice bearing EGFR or Ramos xenografts were injected with three µg [^99m^Tc]Tc-(HE)_3_-E01 (4 MBq, 24.4 MBq/nmol) or three µg of [^123^I]I-(HE)_3_-E01-PIB (1.7 MBq, 10.4 MBq/nmol) and imaged at four h post-injection (p.i.). Images were obtained for 30 min and saved in a matrix with dimensions of 1024 × 256 pixels. The outlines of animals were obtained from digital pictures and overlaid onto the gamma-camera image to facilitate understanding. To estimate imaging contrast, the regions of interest (ROIs) were drawn over EGFR-positive A-431 xenografts, connecting the points with 50% of maximum uptake. Then the ROIs were copied to the kidneys, liver, and the contralateral sites. The number of counts in ROIs was used to calculate tumor-kidney, tumor-to-liver, and tumor-to-contralateral site ratios.

## 5. Conclusions

This study demonstrated that both [^99m^Tc]Tc-(HE)_3_-E01 and [^123^I]I-(HE)_3_-E01-PIB bind specifically and with high affinity to EGFR-expressing cancer cells. The tumor uptake of [^99m^Tc]Tc-(HE)_3_-E01 and [^123^I]I-(HE)_3_-E01-PIB did not differ significantly in the murine model. However, [^123^I]I-(HE)_3_-E01-PIB provided better tumor-to-organ ratios compared to [^99m^Tc]Tc-(HE)_3_-E01. [^123^I]I-(HE)_3_-E01-PIB is a promising agent for imaging of EGFR expression a few hours after injection.

## Figures and Tables

**Table 1:** Characteristics of radiolabeled (HE)_3_-E01 variants.

Variant	Radiochemical Yield, %	Isolated Yield, %	Radiochemical Purity, %	Molar Activity, MBq/nmol
[^99m^Tc]Tc-(HE)_3_-E01	90 ± 2	86 ± 2	99 ± 1	18.0
[^123^I]I-PIB	85 ± 6	n/a	n/a	21.3
[^123^I]I-(HE)_3_-E01-PIB	15 ± 6	12 ± 3	98 ± 2	5.5
[^123^I]I-(HE)_3_-E01	83 ± 1	74 ± 3	100 ± 0	9.2

## Data Availability

The data generated during the current study are available in this paper or the Supplemental Material.

## Supplementary Materials

The following supporting information can be downloaded at: https://www.mdpi.com/article/10.3390/ijms262110609/s1.
