# Supplementary material for: Comparative Preclinical Evaluation of the Tumor-Targeting Properties of Radioiodine and Technetium-Labeled Designed Ankyrin Repeat Proteins for Imaging of Epidermal Growth Factor Receptor Expression in Malignant Tumors
Source: Int J Mol Sci. 2025 Oct 31;26(21):10609. doi: 10.3390/ijms262110609 (PMC12609441; doi:10.3390/ijms262110609)
Supplement: Supplementary file 1 [file ijms-26-10609-s001.zip › ijms-3872329-supplementary.pdf]

Comparative preclinical evaluation of the tumor-targeting properties of radioiodine and technetium-labeled designed ankyrin repeat proteins for imaging of EGFR expression in cancer

Mariia S. Larkina [1,2], Gleb Yanovich [1,2], Lutfi A. Hasnowo [3], Ruslan Varvashenya [1], Feruza Yuldasheva [1], Maria Tretyakova [1], Evgenii Plotnikov [1], Roman Zelchan [1,4], Alexey Schulga [1,5], Elena Konovalova [1,5], Rustam Ziganshin [6], Mikhail Belousov [1,2], Vladimir Tolmachev [7]* and Sergey Deyev [1,5]

[1] Research Centrum for Oncotheranostics, Research School of Chemistry and Applied Biomedical Sciences, Tomsk Polytechnic University, 634050 Tomsk, Russia

[2] Department of Pharmaceutical Analysis, Siberian State Medical University, 634050 Tomsk, Russia

[3] Polytechnic Institute of Nuclear Technology, National Research and Innovation Agency of Indonesia, Yogyakarta 55281, Indonesia

[4] Department of Nuclear Medicine, Cancer Research Institute, Tomsk National Research Medical Center, Russian Academy of Sciences, 634009 Tomsk, Russia

[5] Molecular Immunology Laboratory, Shemyakin & Ovchinnikov Institute of Bioorganic Chemistry, Russian Academy of Sciences, 117997 Moscow, Russia

[6] Collective Use Center "Bioorganic", Shemyakin & Ovchinnikov Institute of Bioorganic Chemistry, Russian Academy of Sciences, 117997 Moscow, Russia

[7] Department of Immunology, Genetics and Pathology, Uppsala University, 75185 Uppsala, Sweden

* Correspondence: vladimir.tolmachev@igp.uu.se

Supplementary Information

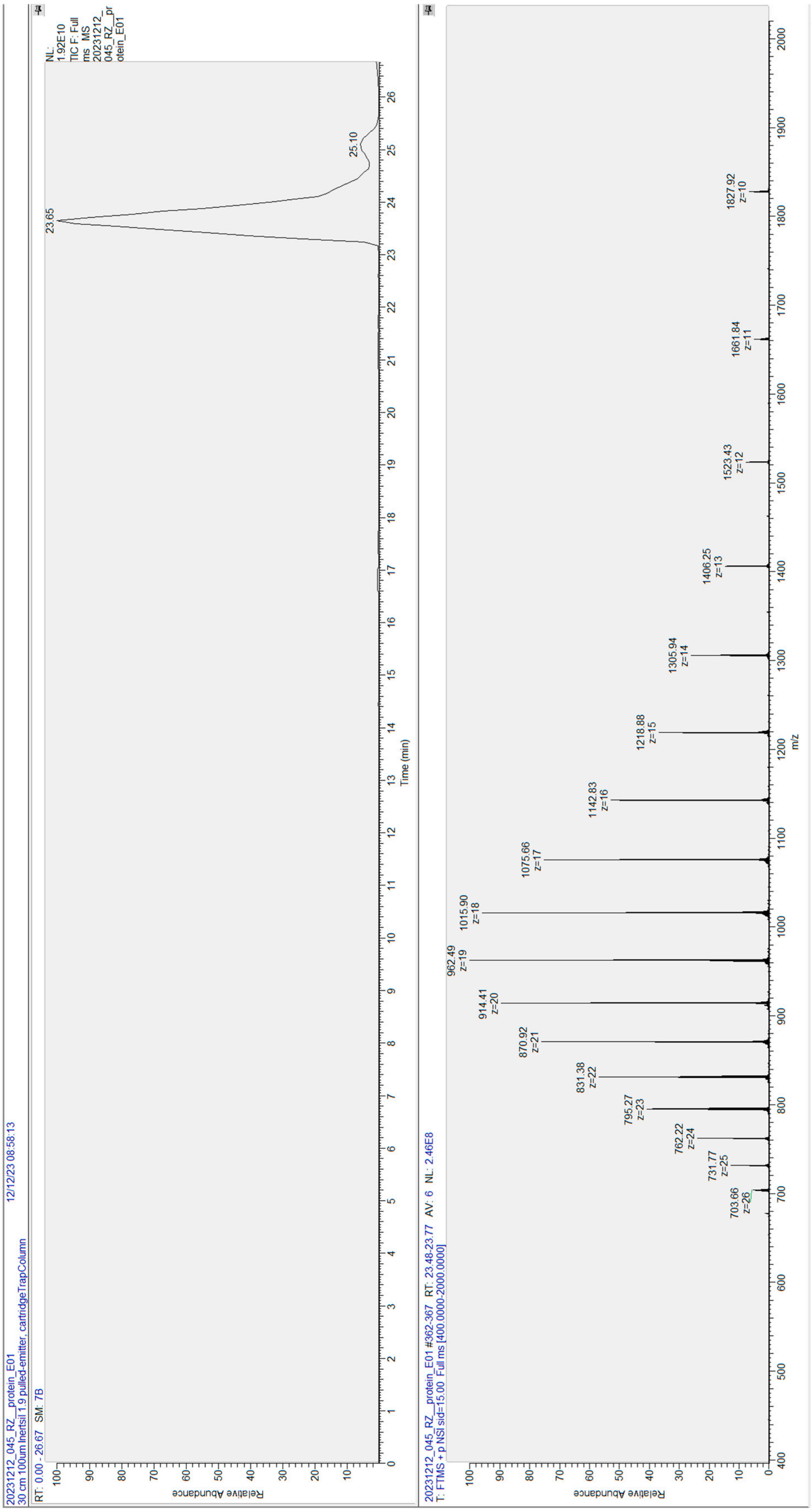

Figure S1. LC-ESI/MS of DARPin E01-(HE)$_3$

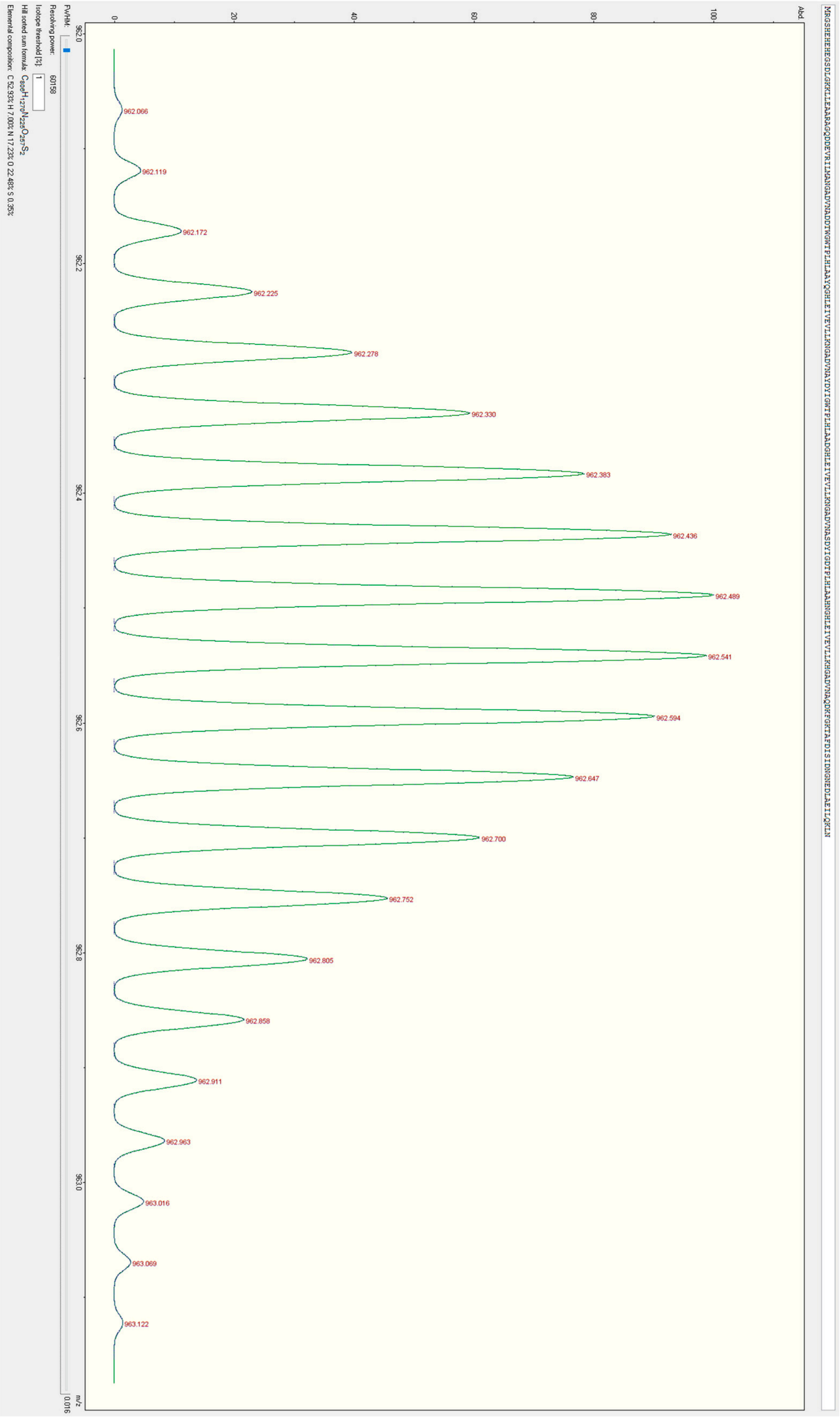

Figure S2. Theoretical high-resolution MS spectra of DARPin E01-(HE)$_3$ at z=19 (Mw$_{calc}$ = 18 260.254 Da)

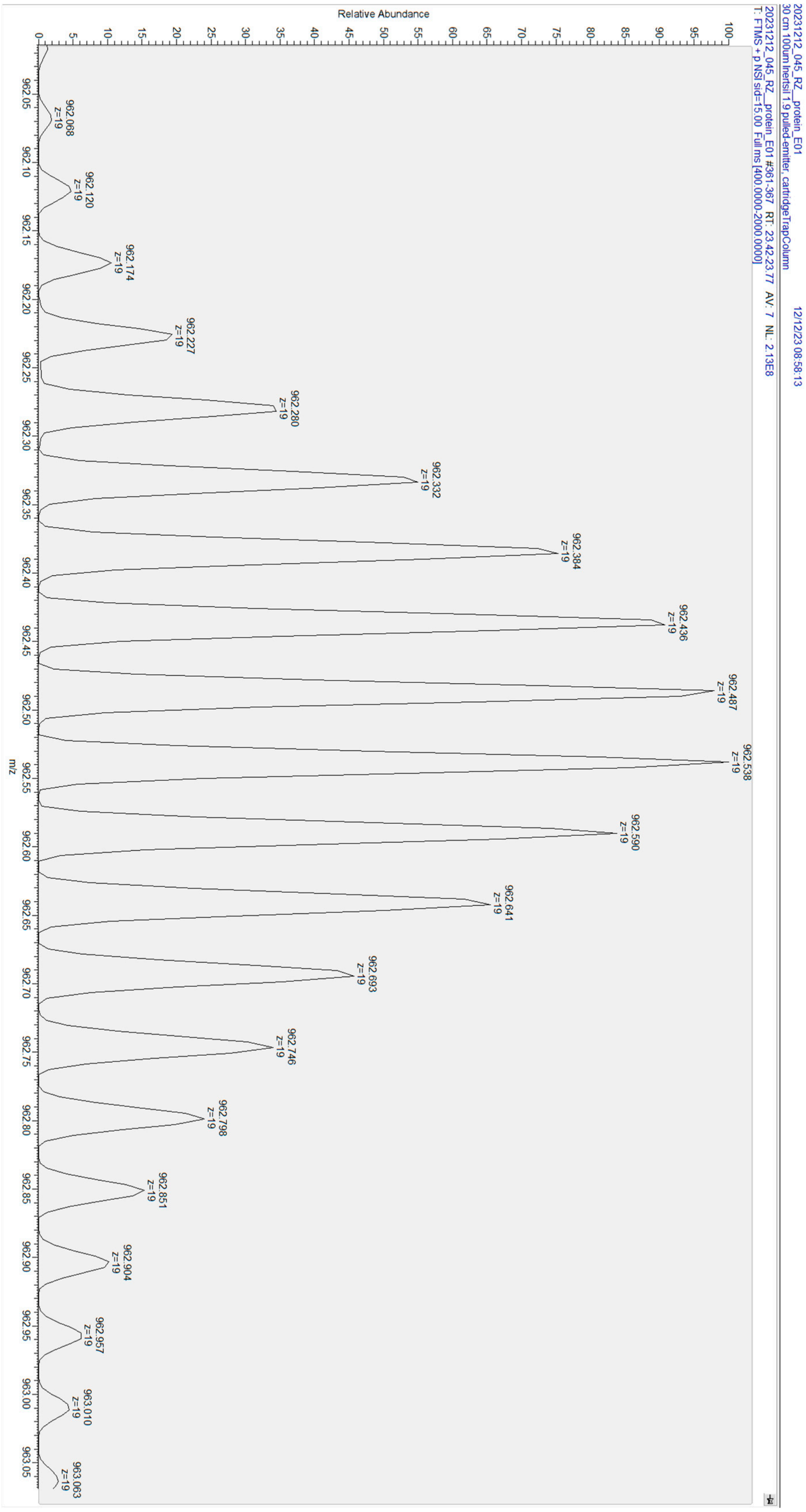

Figure S3. Experimental high-resolution MS spectra of DARPin E01-(HE)$_3$ at z=19 (Mw_{calc} = 18 260.292 Da)

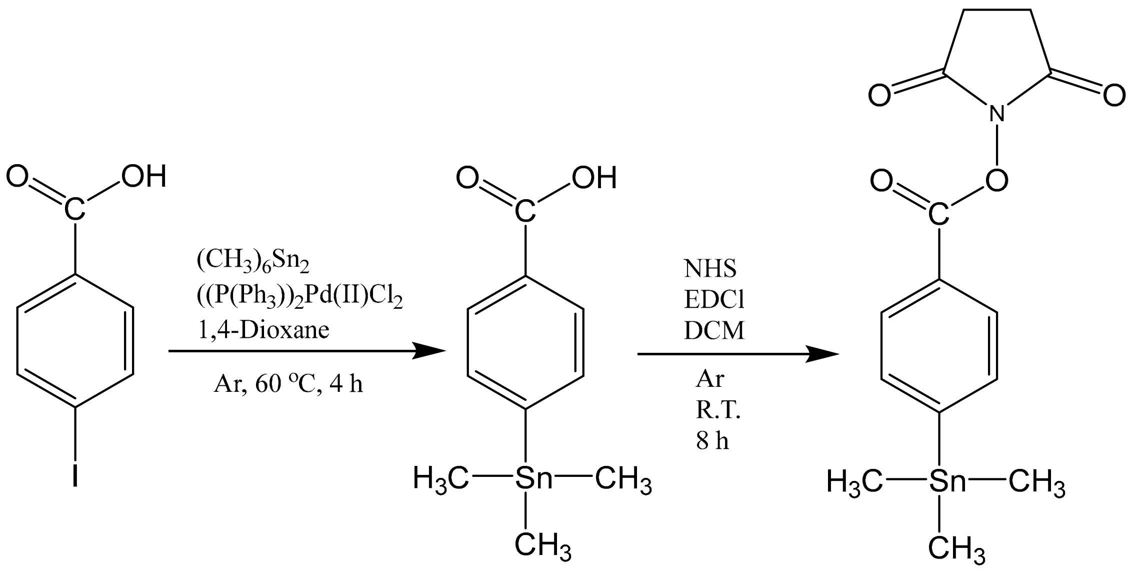

Figure S4. The synthesis scheme of N-succinimidyl-*p*-(trimethylstannyl)benzoate

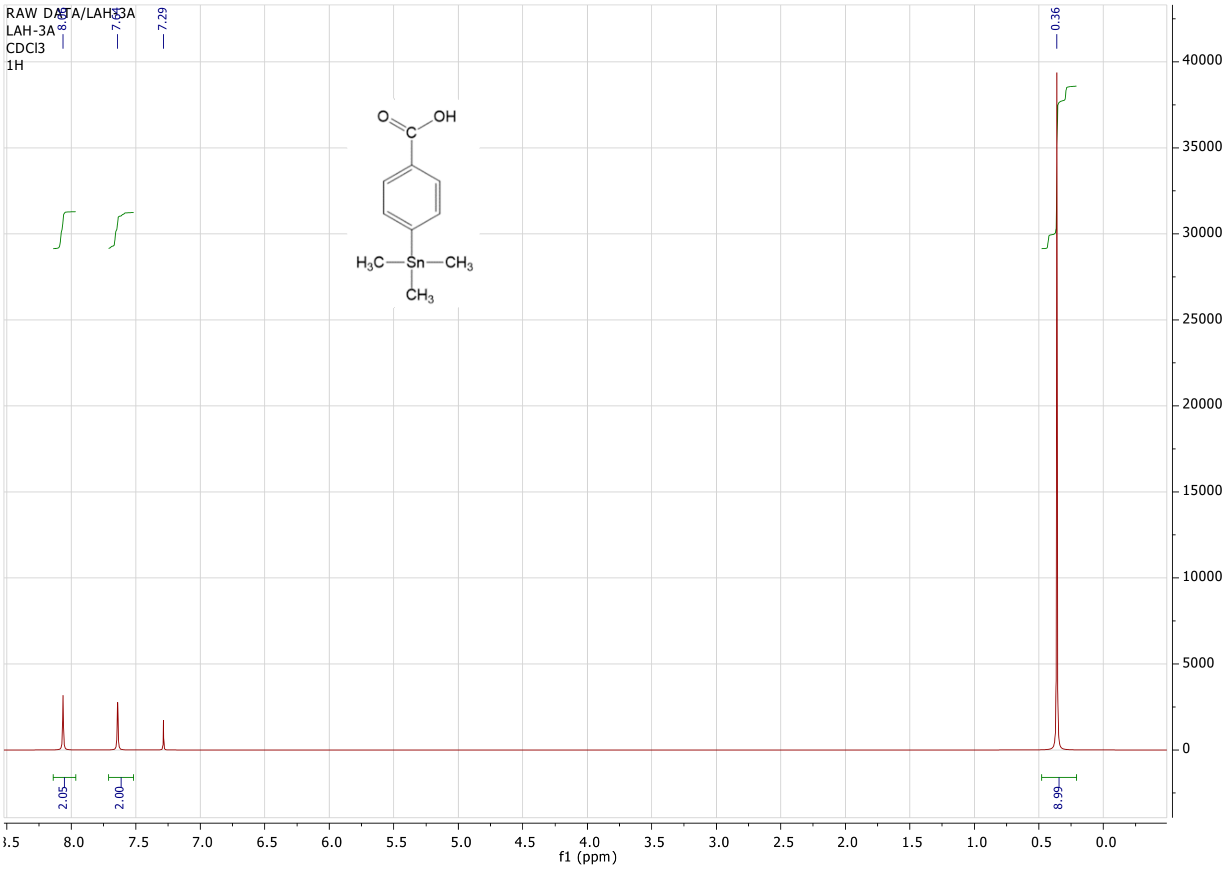

Figure S5. The ^{1}H NMR spectra of *p*-(trimethylstannyl)benzoate

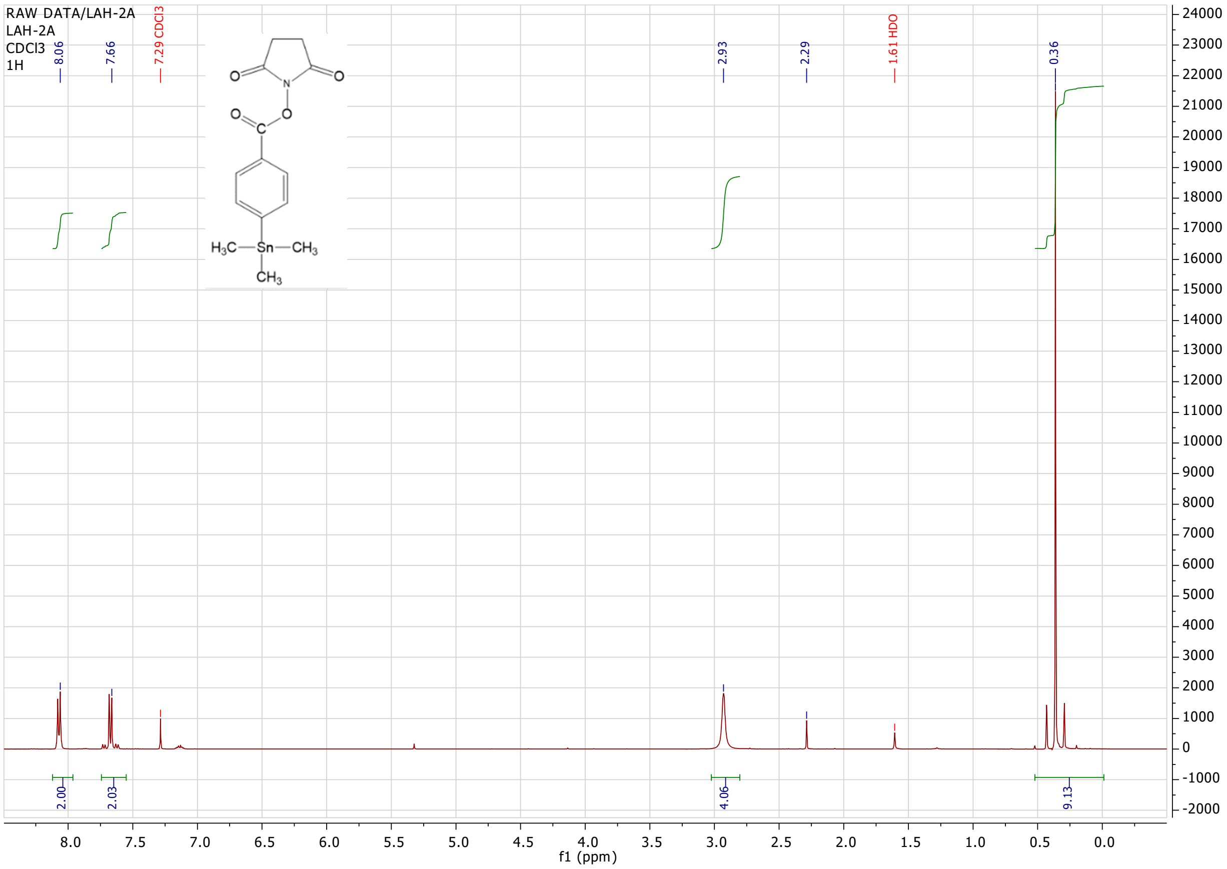

Figure S6. The ^{1}H NMR spectra of N-succinimidyl-p-(trimethylstannyl)benzoate

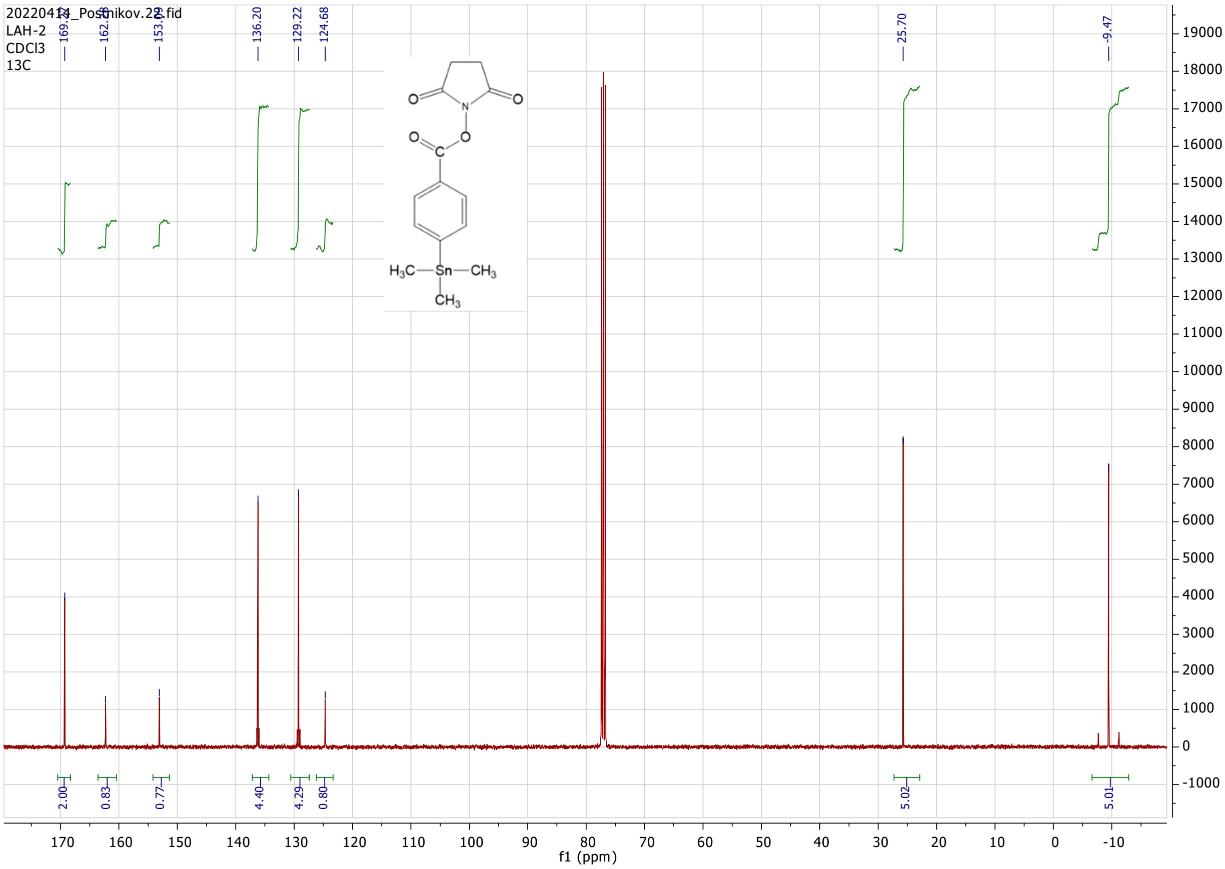

Figure S7. The ^{13}C NMR spectra of N-succinimidyl-*p*-(trimethylstannyl)benzoat

Table S1. Comparative biodistribution of [^{99m}Tc]Tc-(HE)$_3$-E01 variant in CD1 mice 4 h and 24 h post-injection (p.i.), 3 ug or 80 ug per mouse. Data are presented as mean ± SD for four mice. Data for the rest of the GI tract with content and the rest of the body are presented as %ID per whole sample. One-way ANOVA with Bonferroni's multiple comparisons test was performed to find significant differences between time points and doses.

Tissue	3 ug		80 ug	
	4 h	24 h	4 h	24 h
**Blood**	0.3 ± 0.1 [a]	0.13 ±0.01	0.23 ± 0.03 [c]	0.13 ± 0.05
**Salivary glands**	2.6 ± 0.7 [b]	1.9 ± 0.3	3.8 ± 0.3 [c]	2.2 ± 0.5
**Lung**	1.4 ± 0.3	1.1 ± 0.4	1.6 ± 0.3	1.0 ± 0.3
**Liver**	24.3 ± 5.6 [a]	15.6 ± 3.8	19.4 ± 2.2 [c]	11.9 ± 2.8
**Spleen**	7.5 ± 1.8	5,2 ± 1,6	7,3 ± 1,4 [c]	4,1 ±1,5
**Small intestine**	2.0 ± 0,5 [a]	1.2 ± 0.1	2.6 ± 0.4 [c]	1.3 ± 0.1
**Stomach**	1.9 ± 0.6	1.2 ± 0.4	2.6 ± 0.7 [c]	1.3 ± 0.4
**Kidney**	40 ± 9 [b]	29 ± 3	66 ± 14 [c]	35 ± 5
**Muscle**	0.30 ± 0.03 [b]	0.27 ±0.05	0.38 ± 0.04 [c]	0.23 ± 0.02
**Bone**	2.5 ± 0.7	1.7 ± 0.2	2.0 ± 0.5	1.4 ± 0.4
**GI tract with content**	3.3 ± 0.4 [a]	2.0 ± 0.1	4.4 ± 0.8	2.7 ± 0.4
**Rest of the body**	11.0 ± 2.1 [a]	7.2 ± 0.4	14.2 ± 2.9 [c]	7.2 ± 0.5

[a] Significant difference ($p < 0.05$) between [^{99m}Tc]Tc-(HE)$_3$-E01 (3 μg) 4h p.i. and [^{99m}Tc]Tc-(HE)$_3$-E01 (3 μg) 24h p.i. (one-way ANOVA).
[b] Significant difference ($p < 0.05$) between [^{99m}Tc]Tc-(HE)$_3$-E01 (3 μg) 4h p.i. and [^{99m}Tc]Tc-(HE)$_3$-E01 (80 μg) 4h p.i. (one-way ANOVA).
[c] Significant difference ($p < 0.05$) between [^{99m}Tc]Tc-(HE)$_3$-E01 (80 μg) 4h p.i. and [^{99m}Tc]Tc-(HE)$_3$-E01 (80 μg) 24h p.i. (one-way ANOVA).

Table S2. Comparative biodistribution of [^{99m}Tc]Tc-(HE)$_3$-E01 and [^{123}I]I-(HE)3-E01-PIB variants in Nu/j mice bearing EGFR-expressing A-431 and EGFR-non-expressing Ramos xenografts 4 h post-injection (p.i.). Data are presented as mean ± SD for four mice. Data for the rest of the GI tract with contents and the rest of the body are presented as %ID per whole sample.

Tissue	[^{99m}Tc]Tc-(HE)$_3$-E01		[^{123}I]I-(HE)$_3$-E01-PIB	
	A-431	Ramos	A-431	Ramos
**Blood**	0.44 ± 0.03	0.38 ± 0.05	0.4 ± 0.2	0.39 ± 0.04
**Salivary glands**	3.2 ± 0.2 [a]	2.8 ± 0.3	0.23 ± 0.12	0.21 ± 0.04
**Lung**	2.1 ± 0.3 [a]	1.8 ± 0.5	0.3 ± 0.2	0.31 ± 0.02
**Liver**	26.5 ± 4.8 [a]	26.2 ± 3.3	0.9 ± 0.7	1.02 ± 0.03
**Spleen**	7 ± 1 [a]	6.9 ± 1.2	0.2 ± 0.1	0.2 ± 0.1
**Small intestine**	2.8 ± 1.1 [a]	2.1 ± 0.7	0.3 ± 0.2	0.4 ± 0.1
**Stomach**	2.5 ± 0.1 [a,b]	2.0 ± 0.2	0.2 ± 0.1	0.6 ± 0.3
**Kidney**	57 ± 7 [a]	52 ± 5	8.0 ± 3.6	10.6 ± 1.6
**Tumour**	1.17 ± 0.04 [b]	0.45 ± 0.07	1.29 ± 0.36 [c]	0.4 ± 0.1
**Muscle**	0.36 ± 0.05 [a]	0.37 ± 0.09	0.1 ± 0.1	0.08 ± 0.02
**Bone**	3.6 ± 0.9 [a]	3.4 ±1.2	0.2 ± 0.1	0.3 ± 0.1
**GI tract with content**	4.8 ± 1.4	3.5 ± 0.4	4.7 ± 1.6 [c]	8.8 ± 0.6
**Rest of the body**	12.2 ± 1.5 [a]	11.5 ± 1.1	3.1 ± 0.8	3.8 ± 1.0

[a] Significant difference ($p < 0.05$) between [^{99m}Tc]Tc-(HE)$_3$-E01 (A-431) and [^{123}I]I-(HE)$_3$-E01-PIB (A-431) (unpaired t-test).
[b] Significant difference ($p < 0.05$) between [^{99m}Tc]Tc-(HE)$_3$-E01 (A-431) and [^{99m}Tc]Tc-(HE)$_3$-E01 (Ramos) (unpaired t-test).
[c] Significant difference ($p < 0.05$) between [^{123}I]I-(HE)$_3$-E01-PIB (A-431) and [^{123}I]I-(HE)$_3$-E01-PIB (Ramos) (unpaired t-test).

Table S3.Tumor-to-organ ratios of [^{99m}Tc]Tc-(HE)$_3$-E01 and of [^{123}I]I-(HE)$_3$-E01-PIB variants in Nu/J mice bearing A-431 xenografts at 4 h p.i. Data are presented as mean ± SD for four mice.

Tissue	[^{99m}Tc]Tc-(HE)$_3$-E01	[^{123}I]I-(HE)$_3$-E01-PIB
**Blood**	1.2 ± 1.1 ^{a}	3.2 ± 3.0
**Salivary glands**	0.16 ± 0.11 ^{a}	5.5 ± 2.9
**Lung**	0.25 ± 0.14 ^{a}	4.2 ± 6.6
**Liver**	0.02 ± 0.02 ^{a}	1.4 ± 0.5
**Spleen**	0.07 ± 0.06 ^{a}	7.3 ± 2.5
**Small intestine**	0.2 ± 0.1^a	4.6 ± 1.4
**Stomach**	0.2 ± 0.3 ^{a}	5.6 ± 0.5
**Kidney**	0.01 ± 0.01 ^{a}	0.2 ± 0.1
**Muscle**	1.2 ± 0.8 ^{a}	10.9 ± 7.4
**Bone**	0.13 ± 0.06 ^{a}	5.5 ± 1.1

^{a} Significant difference ($p < 0.05$) between [^{99m}Tc]Tc-(HE)$_3$-E01 and [^{123}I]I-(HE)$_3$-E01-PIB (unpaired t-test).
